# Digital Health Revolution in Saudi Arabia: A Narrative Review of Healthcare Transformation

**DOI:** 10.3390/healthcare14172810

**Published:** 2026-09-02

**Authors:** Mahmoud Abdel Hameed Shahin

**Affiliations:** Medical-Surgical and Critical Care Nursing, Nursing Department, Prince Sultan Military College of Health Sciences, Dhahran 34313, Eastern Region, Saudi Arabia; mahmood81us@yahoo.com or mshahein@psmchs.edu.sa; Tel.: +966-507453387

**Keywords:** digital transformation, digital health, healthcare quality, telemedicine, health information systems, Saudi Arabia, Vision 2030

## Abstract

**Highlights:**

**What are the main findings?**

- Digital transformation in Saudi healthcare, driven by Vision 2030, is advancing through EHRs, telemedicine, automation, and data analytics, which collectively enhance care quality, patient safety, and system efficiency when properly governed.
- Successful DT depends on core pillars identified in existing frameworks—technology infrastructure, redesigned clinical processes, data governance, stakeholder engagement, and organizational resilience—yet Saudi Arabia still faces challenges such as fragmented systems, interoperability gaps, and workforce resistance.

**What are the implications of the main findings?**

- Realizing the full benefits of DT in Saudi healthcare requires context-sensitive implementation, strong leadership and governance, sustained investment in technical and human capabilities, and clear regulatory and financing support.
- Addressing infrastructure, interoperability, digital literacy, and change-management barriers is essential for DT to consistently reduce errors, improve outcomes, and deliver long-term economic value across diverse Saudi healthcare settings.

**Abstract:**

Digital transformation (DT) is a strategic priority for healthcare systems seeking to improve the quality of care, patient safety, and operational efficiency. In Saudi Arabia, DT is central to Vision 2030; however, evidence regarding its effects on healthcare quality and safety remains dispersed across technologies and healthcare settings. This narrative review aimed to examine the impact of digital transformation on healthcare quality and patient safety in Saudi Arabia and to identify the principal implementation facilitators, barriers, and policy implications. A structured narrative literature search was conducted for publications from January 2010 to March 2026 using PubMed/MEDLINE, Scopus, Web of Science, Google Scholar, the Saudi Digital Library, and relevant Saudi governmental and policy documents. In total, 64 peer-reviewed studies, reviews, reports, and policy documents addressing digital health initiatives and outcomes related to quality, safety, efficiency, or patient experience were included. Evidence was synthesized thematically, focusing on electronic health records, digital prescribing, online appointment systems, clinical and pharmacy automation, claims and financial analytics, telemedicine, governance, and workforce factors. The reviewed evidence indicates that Electronic Health Record (EHRs), computerized provider order entry, e-prescribing, barcode-supported medication systems, telemedicine, digital appointment platforms, and automated workflows can improve access to care, documentation, care coordination, medication safety, workflow efficiency, and patient experience. However, the benefits of DT are constrained by fragmented infrastructure, limited interoperability, workforce resistance, insufficient digital literacy, funding limitations, and unclear or fragmented governance and regulatory arrangements. In conclusion, digital transformation has substantial potential to enhance healthcare quality and patient safety in Saudi Arabia. Achieving these benefits requires context-sensitive implementation, interoperable systems, strong governance, sustained investment in infrastructure and workforce capacity, and effective leadership and change-management strategies aligned with Vision 2030.

## 1. Introduction

Digital transformation (DT) refers to the strategic integration of information and communication technologies to produce fundamental changes in organizational processes, service delivery, and value creation [1]. In healthcare, DT includes the adoption and integration of digital health technologies to improve healthcare delivery, patient experience, clinical decision-making, and system efficiency [2]. Improving the quality of care and ensuring patient safety remain central global priorities, as preventable harm contributes to adverse outcomes, avoidable costs, and reduced public trust in healthcare systems [3].

In healthcare organizations, DT is implemented through interconnected technologies and processes rather than through a single digital intervention. Core components include electronic health records (EHRs) and electronic medical records (EMRs), computerized provider order entry (CPOE), clinical decision support systems, electronic prescribing, online appointment-booking platforms, telemedicine, clinical and pharmacy automation, and health data analytics [4,5]. EMR is a digital version of a patient’s chart within a single healthcare organization, used primarily by providers in that setting for diagnosis and treatment. EHR, by contrast, is a more comprehensive digital record designed to be shared across multiple healthcare organizations and providers, offering a longitudinal view of a patient’s health history across different care settings [6]. EHRs and CPOE can support complete, accessible, and timely clinical documentation; electronic prescribing and barcode-supported medication systems may reduce medication-related errors; automation can improve the speed, consistency, and traceability of laboratory, pharmacy, and administrative workflows; telemedicine and online appointment systems can improve access, continuity of care, and patient engagement [7]. The effectiveness of these technologies depends on interoperability, data governance, cybersecurity, usability, workforce capability, leadership, and alignment with clinical workflows [8,9,10,11,12,13].

Although several international reviews have examined digital transformation in healthcare, most have focused on broad conceptual frameworks, technology adoption, or individual interventions such as EHRs, electronic prescribing, telemedicine, or automation. Consequently, they provide limited guidance on how multiple digital-health technologies interact within a specific national health system to influence healthcare quality and patient safety. In addition, much of the available evidence is derived from high-income Western health systems with different models of care, digital infrastructure, financing arrangements, regulatory environments, workforce composition, and levels of interoperability. These findings cannot be assumed to be directly transferable to Saudi Arabia [4,5,7,14,15].

A Saudi-specific synthesis is needed because digital-health transformation in the Kingdom is occurring within the policy context of Vision 2030 and the Health Sector Transformation Program. Saudi healthcare organizations differ in their stages of digital maturity, public- and private-sector structures, availability of technical infrastructure, interoperability capacity, workforce readiness, digital literacy, data governance requirements, and experience with large-scale implementation of electronic clinical systems. Moreover, Saudi evidence is dispersed across individual technologies and organizational settings, making it difficult for healthcare leaders and policymakers to identify the conditions under which digital transformation improves quality and patient safety [16,17].

Therefore, this narrative review synthesizes international and Saudi Arabian evidence on the effects of digital transformation—including EHRs/EMRs, computerized provider order entry, digital prescribing, telemedicine, online appointment systems, clinical and pharmacy automation, and health-data analytics—on healthcare quality and patient safety. It further identifies Saudi-specific implementation facilitators, barriers, evidence gaps, and policy implications. By integrating technology-specific evidence with organizational and policy considerations, this review provides a context-sensitive perspective to inform digital health planning, implementation, and future research in Saudi healthcare organizations.

## 2. Methodology

This article is a narrative review that aims to synthesize international and Saudi Arabian evidence on digital transformation in healthcare, with a particular focus on quality and patient safety outcomes. The narrative review approach was chosen to allow integration of empirical findings, conceptual frameworks, and policy documents that collectively inform digital health transformation in the Saudi context.

A structured literature search was undertaken between January 2010 and March 2026 in the following electronic databases: PubMed/MEDLINE, Scopus, Web of Science, and Google Scholar. Additional regionally relevant sources were identified through searches of local and regional databases (such as the Saudi Digital Library) and official websites of the Saudi Ministry of Health and related governmental bodies. The search strategy combined keywords and Boolean operators related to digital transformation and healthcare quality and safety, including terms such as “digital transformation”, “digital health”, “health information systems”, “electronic health records”, “telemedicine”, “automation”, “Saudi Arabia”, “quality of care”, and “patient safety”.

To complement database searches, we performed backward and forward citation tracking of key articles and frameworks, and we screened reference lists of relevant publications and national strategy documents (e.g., Vision 2030 and associated digital-health policies) to identify additional academic and grey literature. Search results were first screened by title and abstract for relevance to digital transformation in healthcare, followed by full-text assessment of potentially eligible publications.

Inclusion criteria comprised: (1) peer-reviewed articles, reviews, reports, or policy documents addressing digital health or digital transformation initiatives in healthcare organizations; (2) studies reporting outcomes related to quality of care, patient safety, efficiency, or patient experience; and (3) publications focusing on Saudi Arabia or health systems with comparable digital-health priorities, as well as influential international frameworks applicable to the Saudi context. We excluded studies that described only technical system specifications without clinical or organizational implications, purely editorial commentaries without empirical or conceptual contributions, and publications not available in English or Arabic.

The structured search identified 612 records from PubMed/MEDLINE, Scopus, Web of Science, Google Scholar, and the Saudi Digital Library. A further 38 records were identified through backward and forward citation tracking, reference list screening, and searches of relevant government and policy websites. After removing 126 duplicate records, 524 unique records underwent title and abstract screening. At this stage, 402 records were excluded because they were not related to digital transformation in healthcare, did not report outcomes relevant to healthcare quality, patient safety, efficiency, or patient experience, did not meet the publication-type or language criteria, or were outside the scope of the review. The full texts of 122 reports were assessed for eligibility. Following full-text review, 58 reports were excluded because they focused only on technical system specifications without clinical or organizational implications, were editorials or commentaries without relevant empirical or conceptual contributions, were duplicate or less complete reports, were not available in English or Arabic, or did not provide sufficient relevance to the Saudi Arabian or comparable healthcare context. In total, 64 sources were included in the final thematic narrative synthesis.

Given the narrative design, no formal risk-of-bias scoring or quantitative meta-analysis was performed. Instead, the included sources were analyzed thematically, with attention to recurring digital transformation elements (e.g., electronic health records, digital prescribing, telemedicine, automation, and governance), and their reported impacts on healthcare quality and safety in Saudi Arabia and internationally. This approach provides a structured yet flexible synthesis that supports interpretation of current evidence and identification of contextual implementation considerations for Saudi healthcare organizations.

### Search Strategy

A structured literature search was conducted between January 2010 and March 2026 in PubMed/MEDLINE, Scopus, Web of Science, Google Scholar, and the Saudi Digital Library. Additional relevant policy documents and grey literature were identified through the websites of the Saudi Ministry of Health, the Saudi Food and Drug Authority, and other relevant governmental bodies.

The search strategy was structured around four concept groups: (1) digital transformation and digital-health technologies; (2) healthcare settings; (3) healthcare-quality and patient-safety outcomes; and (4) Saudi Arabia or comparable health-system contexts. Synonyms within each concept group were combined using the Boolean operator OR, and concept groups were combined using AND. Truncation symbols and phrase searching were adapted to each database’s syntax.

The principal search string was:

(“digital transformation” OR “digital health” OR “health information system*” OR “electronic health record*” OR EHR OR EMR OR “electronic medical record*” OR “computerized provider order entry” OR CPOE OR “clinical decision support” OR “electronic prescrib*” OR e-prescrib* OR telemedicine OR telehealth OR automation OR “robotic process automation” OR “automated dispensing cabinet*” OR “online appointment*” OR “patient portal*” OR “health data analytic*”) AND (healthcare OR “health care” OR hospital* OR clinic* OR “primary care”) AND (“quality of care” OR “healthcare quality” OR “patient safety” OR “medication error*” OR “clinical outcome*” OR efficiency OR “patient experience” OR “patient satisfaction”) AND (“Saudi Arabia” OR Saudi OR “Gulf Cooperation Council” OR GCC).

To ensure that influential international frameworks and evidence relevant to Saudi healthcare were not missed, an additional broader search was conducted without the location filter:

(digital-health technology terms) AND (healthcare-setting terms) AND (quality-and-safety outcome terms).

Database filters were applied where available: publication date from January 2010 to March 2026, English or Arabic language, and article, review, report, or policy-document publication type. Backward and forward citation searches of relevant reviews, key empirical studies, and national digital-health policy documents supplemented the electronic database searches. The list of empirical studies included in the narrative review is provided as Supplementary Data (Table S1).

## 3. Review of the Recent Literature About Digital Transformation in Healthcare

### 3.1. Digital Transformation in Healthcare Organizations

DT has been recognized as a strategic priority for healthcare organizations seeking to improve operational efficiency, clinical outcomes, and financial sustainability [9,12]. Healthcare institutions have increasingly adopted EHRs, telemedicine platforms, and artificial intelligence (AI) to optimize clinical processes and enable data-driven decision-making [9,12]. However, the pace and impact of DT differ substantially across settings, influenced by organizational culture, regulatory environments, and resource availability [11].

In this section, key definitions and conceptual frameworks are outlined; the organizational dimensions of DT are discussed; and empirical evidence on its quality and safety outcomes is summarized.

#### 3.1.1. Definitions and Frameworks

DT in healthcare extends beyond converting paper records into electronic formats. It involves changes in clinical workflows, organizational structures, patient engagement, data governance, and decision-making processes enabled by digital technologies. A healthcare-specific framework proposed by Dal Mas et al. identifies technology infrastructure, clinical-process redesign, data governance, and stakeholder engagement as interrelated dimensions of successful transformation [9]. User-centered design, interoperability, privacy protection, workforce capability, and leadership are also consistently identified as implementation requirements in digital-health literature. However, the practical application of these principles must be adapted to the regulatory, organizational, and technological conditions of individual health systems, including Saudi Arabia.

Dal Mas et al. [9] proposed an interdisciplinary model comprising four pillars: technology infrastructure, clinical process redesign, data governance, and stakeholder engagement. These pillars function as interconnected components linked by continuous feedback loops, allowing policies and workflows to adapt as technologies mature. The model emphasizes that investment in hardware or software alone is insufficient; robust data policies and active engagement of clinicians and patients are essential [9].

Drawing on socio-technical systems theory, Frisinger and Papachristou [10] developed a model with three reinforcing levels: user-centered design, interoperability standards, and governance mechanisms. They stressed that digital solutions are unlikely to deliver the anticipated quality and safety benefits if clinician and patient feedback are not incorporated into design and implementation. Tortorella et al. [13] focused on organizational resilience in the context of healthcare 4.0, identifying operational, technical, and organizational resilience—captured in process flexibility, system redundancy, and leadership agility—as key dimensions. According to their model, DT should enhance not only efficiency but also hospitals’ capacity to respond to shocks such as pandemics or cyberattacks.

Across these paradigms, strong leadership commitment, effective data governance, and sustained user engagement emerge as key facilitators, whereas legacy IT systems, regulatory constraints, and workforce resistance constitute common barriers [11,12]. Kakale’s [11] systematic review underscored the need for contextual adaptation when applying generic frameworks to specific healthcare systems. For example, data governance practices in European hospitals require significant modification to comply with Saudi privacy regulations and patient-consent conventions.

Methodological perspectives also shape DT evaluation. Ghanad [18] emphasized the importance of clearly defined evaluation criteria and key performance indicators (KPIs) for assessing digital initiatives; without robust metrics, organizations risk implementing technologies that cannot be systematically evaluated. Vărzaru and Bocean [19] further demonstrated links between DT and sustainable economic development, arguing that alignment with national policy objectives improves access to funding and stimulates innovation. Synthesizing these insights, an enhanced model for Saudi healthcare would integrate the structural pillars of Dal Mas et al. [9], the user-centered processes of Frisinger and Papachristou [10], the resilience criteria of Tortorella et al. [13], and the contextual-sensitivity framework of Kakale [11], all supported by measurable evaluation mechanisms.

#### 3.1.2. Digital Transformation and Organizational Change

Successful DT depends on organizational change processes, with leadership, culture, and workforce capabilities playing central roles. Strong executive sponsorship is critical for articulating vision, securing resources, and modeling digital behaviors that build momentum [9]. Leadership teams that are digitally and technologically literate are better positioned to bridge gaps between frontline staff and IT functions [10]. Tortorella et al. [13] identified leadership agility as a key pillar of organizational resilience, showing that rapid decision-making and the empowerment of middle managers support adaptation when digital initiatives encounter unforeseen obstacles.

Digital readiness extends beyond technical training to encompass openness to change and commitment to lifelong learning [9]. Resistance to DT is often driven by fears of job loss and difficulties in adapting to new work practices, which can be mitigated through structured change-management programs, including peer-led workshops, digital champions, and support forums [9]. Vărzaru and Bocean [19] found that staff participation in co-design sessions for telehealth implementation increased satisfaction scores by 20% and accelerated adoption. Integrating digital literacy into professional development plans helps ensure that employees across all levels possess the competencies required to use emerging tools effectively.

At the macro level, alignment with national e-health strategies and regulatory frameworks provides a critical foundation. Saudi Arabia’s Vision 2030, which emphasizes sustainable economic development and digital infrastructure, creates incentives for hospitals to modernize systems [20]. Dal Mas et al. [9] highlighted the importance of robust data-governance policies, including interoperability standards, cybersecurity protocols, and privacy safeguards, to maintain public trust and regulatory compliance. Multidisciplinary governance committees ensure that ethical and legal considerations are embedded throughout the digital project lifecycle, from procurement to post-deployment [9,10].

#### 3.1.3. Digital Transformation and Outcomes for Quality and Safety

Much of the evidence summarized in this section originates from health systems outside Saudi Arabia. Although these studies identify potentially relevant mechanisms and implementation considerations, their reported effects should not be assumed to apply directly to Saudi healthcare organizations because digital maturity, interoperability, workforce capacity, governance, financing, patient populations, and clinical workflows differ across contexts. Saudi-specific evaluation is therefore needed before international findings are translated into national policy or scaled implementation.

Evidence indicates that digital health interventions can improve selected dimensions of healthcare quality and patient safety when integrated into clinical workflows and supported by appropriate governance and training. EHRs, computerized provider order entry, and clinical decision-support systems can improve the availability and completeness of clinical information, facilitate communication among healthcare professionals, and support medication-safety processes [8,21,22,23,24,25,26]. Electronic prescribing has been associated with fewer medication and prescribing errors, although system usability problems and new forms of electronic prescribing errors may still occur [27,28,29,30,31,32,33,34].

Digital technologies may also improve access and service efficiency. Patient portals, online appointment systems, and telemedicine can support communication, follow-up, and access to care, while automation may improve the efficiency of laboratory, pharmacy, and administrative workflows [35,36,37,38,39]. Nevertheless, the magnitude and consistency of these effects vary by technology, setting, implementation quality, staff training, interoperability, and the outcomes assessed. Therefore, digital transformation should be interpreted as an organizational and socio-technical process rather than as a single intervention with uniform effects.

### 3.2. Elements of Digital Transformation in Healthcare Organizations and Its Effect on Healthcare Quality and Safety

DT in healthcare is driven by several core elements: technology infrastructure, redesigned clinical processes, data governance, stakeholder engagement, and leadership and culture. These elements collectively determine an organization’s capacity to adopt, embed, and sustain digital innovations that improve quality, safety, and patient experience [9]. DT may contribute to improved healthcare quality and patient safety by enhancing data accessibility, reducing medication errors, and supporting clinical decision-making through systems such as EHRs, CPOE, and clinical decision support systems (CDSSs), especially when they are appropriately implemented and supported by interoperability, data quality, training, governance, and workflow integration. Key factors influencing these outcomes include system integration, data completeness, user adoption, interoperability, and the presence of safety features such as barcode medication administration and real-time alert systems [40].

Although international evidence provides useful implementation lessons, Saudi-specific studies are required to establish the intervention’s effectiveness, safety, feasibility, equity, and cost implications within local healthcare settings. In Saudi Arabia, substantial investments aligned with Vision 2030 have accelerated digital health adoption; however, variability in system utilization, incomplete clinical data entry, and limited implementation of advanced safety tools continue to affect optimal outcomes [7]. While digital platforms have improved documentation and workflow efficiency, gaps in user engagement and in the integration of system functionality remain critical barriers, underscoring the need for standardized implementation, training, and enforcement of data quality measures to realize improvements in healthcare quality and patient safety fully.

#### 3.2.1. Electronic Medical Record Systems

Electronic medical records (EMRs) are digital records maintained within a single healthcare facility, whereas EHRs aggregate patient information across multiple care settings to support continuity of care [12]. EMRs constitute a foundational component of DT, enabling structured data collection, standardized workflows, and real-time access to patient information [9]. When appropriately implemented, EMRs improve clinical decision-making, reduce diagnostic testing duplication, and provide reliable audit trails for compliance and quality management [12].

EMRs consolidate fragmented information—laboratory results, medication lists, and clinical notes—into a single, authorized source, thereby reducing transcription errors and loss of paper-based documentation [12]. Hospitals with mature EMR systems have demonstrated a 20% decrease in medication errors and a 15% increase in care coordination, reflecting improved patient safety [12]. Embedded clinical decision support (CDS) modules generate alerts regarding critical laboratory values, allergies, and guideline deviations, transforming raw data into actionable clinical knowledge [9]. EMRs also enable downstream analytics for retrospective studies, population health management, and predictive modeling, which are central to value-based care [25].

International and regional evidence converges on the importance of EHRs as a core DT factor for improving quality and safety. EHRs support evidence-based decision-making and streamline workflows by enhancing communication among providers and facilitating access to accurate patient information [23,24,26]. Atasoy et al. [21] found that EHRs enhanced patient safety by integrating CDS tools that prevented adverse drug events and supported protocol adherence. Ayaad et al. [22] reported that hospitals using EMRs achieved higher overall service quality than comparable institutions relying on paper charts, with improvements in diagnostic efficiency, documentation coherence, and patient satisfaction.

Systematic reviews indicate that EMRs improve real-time data access, strengthen care coordination, and expand the use of CDS, including reminders and alerts [25,41]. Upadhyay and Hu [24] highlighted structured documentation and reminder functions as key mechanisms through which EHRs support patient safety.

Nevertheless, EMR implementations face challenges. Interoperability remains a key concern, as legacy systems and proprietary data formats often fail to communicate effectively, forcing clinicians to navigate multiple interfaces or manually reconcile information [9]. Poor workflow integration can slow documentation and disrupt patient flow. Learning curves are steep, and resistance to change is common; without comprehensive training and support, clinicians may revert to paper-based workarounds that undermine data integrity and ROI [11].

In a concurrent mixed-methods study conducted by Alghamdi and Sayed [42] across five public hospitals in Riyadh, involving 61 hospital leaders involved in EMR implementation, the findings revealed that most leaders (80.3%) and staff (88.5%) reported positive perceptions of EMR use. Regression analysis identified adequate infrastructure (*p* = 0.003) and staff perception (*p* = 0.002) as significant predictors of successful implementation. Key challenges included training and adoption, system-related issues, and time constraints, while recommended improvements focused on user training, system enhancements, and greater user involvement. The findings highlight the critical role of leadership and the need for coordinated efforts to optimize EMR implementation. Interoperability issues prompted the formation of a multidisciplinary governance committee that standardized consent procedures and data-access policies, thereby enhancing clinician confidence in EMR reliability [10].

Over time, EMRs were interfaced with patient portals and mobile health applications to improve patient engagement and self-management [10]. Application programming interfaces (APIs) enabled data exchange with third-party applications, advancing the vision of a fully interoperable digital health ecosystem [19].

#### 3.2.2. Digital Prescribing Systems

Electronic prescribing (e-prescribing) platforms, also known as digital prescribing systems (DPS), enable clinicians to create, exchange, and manage medication orders electronically, eliminating handwritten or faxed prescriptions [12]. Advanced e-prescribing systems integrate with pharmacy information systems and CDS tools to perform real-time checks for allergies, drug interactions, and dosing errors [9]. These systems supplement EMRs by reducing transcription errors, accelerating pharmaceutical processes, and supporting continuity of care [12].

E-prescribing centralizes prescription workflows within unified platforms that auto-populate patient demographics, allergy histories, and formulary preferences [12]. Integrated CDS modules alert clinicians to high-risk medication combinations and dosing errors, reducing medication errors [9]. Implementation has been associated with a 25% reduction in prescribing errors and a 20% decrease in pharmacy processing times [12]. Audit trails documenting prescription modifications increase transparency and support safety investigations, while electronic formulary checks improve regulatory and insurance compliance [9]. Automated renewal requests and refill reminders enhance patient engagement and adherence.

Porterfield et al. [31] and Roumeliotis et al. [32] reported that e-prescribing significantly reduced prescribing errors and improved medication management. In a systematic review and meta-analysis, Roumeliotis et al. [32] found that e-prescribing reduced medication errors by 76%, dosing errors by 83%, and other medication-related errors by 52%. In the English National Health Service, implementing prescription interfaces was projected to prevent thousands of incorrect dosage prescriptions and save 4–22 lives annually [29].

Cassidy et al. [30] reported that most studies in community pharmacy settings showed decreased prescription errors and improved medication safety following e-prescribing implementation. Workflow efficiency and interprofessional communication also improved, although some studies noted reduced face-to-face interaction [31,34]. Advanced, machine-learning-enabled e-prescribing systems further enhance safety by detecting risks that traditional CDS may miss, with high rates of correct, clinically relevant alerts [33].

Nevertheless, DPS can introduce new error types, including incorrect dropdown selections and incomplete information; approximately 8.5% of electronically prescribed medications in one study required correction [28]. Continuous monitoring and interface optimization are therefore needed.

Despite these advantages, limited interoperability, parallel workflows, and usability challenges can reduce adoption [9,11]. Regulatory requirements for electronic signatures and secure data transmission add complexity. A retrospective study conducted at a tertiary military hospital in Saudi Arabia evaluated the impact of modifications to the electronic prescribing system on inpatient prescribing errors across 29,554 admissions. The overall prescribing error rate significantly decreased from 1.43% to 0.51% (*p* < 0.001), representing a 49.8% reduction. Despite variability of different departments, the findings demonstrate that system modifications can substantially improve medication safety, while highlighting the need for continuous, department-specific optimization [27]. Future-oriented analyses suggest that integrating e-prescribing with mobile health apps and patient-facing portals can further enhance medication adherence [10]. Adoption of interoperability standards such as HL7 FHIR is expected to enable the secure exchange of prescription data across care settings, aligning with Vision 2030’s digital health objectives [43].

#### 3.2.3. Online Appointment Booking Systems

Online appointment systems enable patients to schedule, modify, and cancel appointments via web- or mobile-based interfaces, reducing reliance on telephone scheduling [12]. These platforms integrate calendar management, automated reminders, and patient self-service portals, decreasing administrative burden and empowering patients with real-time access to available slots [9].

Online appointment systems are associated with improved access, reduced waiting times, and lower no-show rates. Evidence indicates that online booking may influence rates, although results differ across healthcare settings and accompanying reminder strategies. Dal Mas et al. [9] reported a 30% reduction in average wait times for routine follow-up visits when patients used self-scheduling tools. Automated SMS and email reminders have been associated with a 25% decline in no-show rates [10]. Analytics derived from booking data enable clinics to anticipate peak demand and adjust staffing to improve patient flow [9].

In Norway, 80% of patients found online scheduling easier and more efficient than phone-based systems, with reduced congestion during peak periods [39]. In China, combining hierarchical medical settings with online reservations ensured that minor conditions were treated at primary levels, while complex cases were referred to higher-level institutions, improving efficiency [44].

Carini et al. [45] reported that patient portals with integrated appointment reminders improved follow-up adherence, while SMS and email reminders increased attendance by up to 67%. Ng’andu and Haabazoka [46] showed that combining EHRs with online appointment systems in Zambia improved patient flow and care coordination, thereby enhancing patient safety. Dendere et al. [47] found that patient portals and digital booking systems reduced double-booking and missing documentation, with positive effects on quality and safety.

For providers, online systems reduce administrative workload, minimize paperwork and data-entry errors, and enable clinicians to review patient history before consultations, improving diagnostic accuracy and treatment planning [39,46,48].

Integration challenges with legacy clinical information systems remain significant. Custom APIs or middleware are often required to synchronize appointment data, and workflow disruptions may occur when online systems fail to reflect real-time changes, such as emergency walk-ins [9,11]. A retrospective study in an ophthalmology practice and university hospital evaluated the impact of online appointment scheduling (OAS) over 20 months, including 16,894 practice and 81,173 hospital appointments. OAS adoption increased over time and was associated with reduced no-show rates in the practice (1.8% vs. 5.9%, *p* < 0.0001), but higher no-show rates in the hospital (14.3% vs. 11.2%, *p* < 0.0001). In the hospital setting, SMS reminders and regular consultations were effective in reducing no-shows (ORs = 0.93 and 0.40, respectively). Additionally, OAS improved appointment utilization in practice by significantly reducing unused and unbooked slots, highlighting its role in enhancing efficiency while underscoring the need for complementary reminder strategies in larger healthcare settings [37]. Future enhancements include AI-driven predictive scheduling to prioritize high-risk patients, dynamic rescheduling, and integration with mobile health applications and telemedicine link generation [13,19].

#### 3.2.4. Automation of Clinical Tasks

Automation in healthcare includes technologies that support repetitive, rule-based clinical and administrative activities, such as laboratory automation, electronic data processing, automated patient feedback systems, and selected artificial-intelligence applications. Evidence from clinical laboratory settings suggests that total laboratory automation can improve throughput and reduce manual handling and processing errors. Al Naam et al. [49] reported that total laboratory automation increased test throughput per worker by 1.4 to 3.7 times and reduced sample-processing errors. In radiation oncology, AI and automation enhanced treatment-planning efficiency, reduced inter-observer variability, and improved treatment precision [50]; however, clinical validation, staff oversight, transparency, and accountability remain essential. Administrative robotic process automation may also reduce processing time and improve workflow efficiency in selected healthcare processes.

In hospital pharmacy, Sharaf [51] found that automated systems reduced medication errors, improved workflow efficiency, and enhanced patient safety. Meknassi Salime et al. [52] highlighted benefits for drug tracking and workload reduction but noted compatibility and training challenges. Administrative automation also improves performance; a Lean Six Sigma project applying RPA to claims verification saved 380 min of processing time and increased process cycle efficiency from 69.07% to 95.54% [53].

Automated patient-reported experience measures (PREMs) increased feedback responses tenfold in one hospital, providing richer data for quality improvement while reducing staff burden [54]. AI-based virtual assistants, such as ChatGPT-3, have shown potential to support documentation, medication suggestions, and patient communication, enhancing productivity and decision-making [36].

The implementation of automation requires attention to system integration, data protection, cybersecurity, validation, workforce training, and clear accountability for automated decisions. Healthcare organizations should therefore introduce automation through phased implementation and continuous evaluation of operational and patient-safety outcomes [51,52,53,54].

#### 3.2.5. Automation of Pharmacy Dispensing Systems

Automation of pharmacy dispensing systems uses technologies such as automated dispensing cabinets (ADCs) and barcode-based verification to streamline medication storage, retrieval, and distribution [12]. ADCs are secure, computer-controlled cabinets located near point-of-care areas, enabling rapid medication access and accurate inventory control [13]. Barcode scanning of wristbands and medication packages ensures compliance with the “five rights” of medication administration, reducing dispensing errors [9].

ADCs linked to pharmacy information systems have yielded a 32% reduction in dispensing errors and a 25% decrease in medication retrieval time [13]. Inventory control modules trigger reorder points, reducing stockouts and expired-drug waste, and data-driven replenishment cuts inventory-related costs by 15% annually [9,10]. In a study that was conducted in intensive care units retrospectively compared prescription, dispensing, and administration medication error rates before and after implementation of ADCs using data from the unit’s medication error reporting system and classifying severity according to National Coordinating Council for Medication Error Reporting and Prevention categories; after ADC adoption, prescription errors fell from 3.03 to 1.75 per 100,000 prescriptions, dispensing errors from 3.87 to 0 per 100,000 dispensations, administration errors from 0.046% to 0.026%, and category B and D errors decreased by 75% with category C errors reduced by 43%, indicating that ADCs substantially improve medication safety in this critical care setting [55].

ADCs, barcode medication administration (BCMA), and electronic medication-management systems have been shown to reduce medication administration errors by approximately 50% or more when integrated with electronic ordering [56,57,58]. A quality-improvement project at King Fahad Armed Forces Hospital found that ADC implementation reduced medication turnaround time by 83%, decreased drug consumption by 24%, and reduced returned medications by 72% [5].

Craswell et al. [59] reported that distributed ADCs improved workflow organization and nurse satisfaction, despite initial adaptation challenges. Zheng et al. [58] highlighted reductions in time spent administering medications and restocking, as well as fewer end-of-shift controlled-drug counts. However, barcode quality issues, interface usability problems, and workarounds—such as inappropriate use of override functions—pose new risks [58,59]. Boyd and Chaffee [57] emphasized the need for financial and operational sustainability, as well as standardized evaluation metrics.

Challenges include high upfront hardware and software costs, integration difficulties, and staff resistance due to perceived loss of autonomy or cumbersome authentication procedures [9,11]. Effective implementations involve stakeholder engagement during planning, phased rollouts, comprehensive training, and clear policies for ADC access and override in emergencies [10,19].

#### 3.2.6. Claims Data, Billing, and Finance Data Systems

Automation of claims, billing, and finance systems replaces paper-based processes with electronic charge capture, claim generation, and exchange with insurers, integrated with clinical documentation [12]. These systems auto-fill billing codes, reduce reimbursement turnaround times, and provide real-time insights into financial performance [9]. They can support quality and safety by enabling performance measurements, early risk detection, and more efficient resource allocation.

Automated coding engines map clinical procedures and diagnoses to classification systems such as ICD-10 and CPT, reducing claim rejections by up to 30% [9]. Real-time eligibility verification prevents billing for non-covered services, and analytics dashboards visualize trends in payer mix, denial rates, and days in accounts receivable [10]. Automated alerts identify high-risk claims for manual review, and electronic remittance advice (ERA) streamlines payment posting, reducing posting time by 50% [9].

Kaiser et al. [60] showed that administrative claims data can be used to monitor mortality, readmissions, patient satisfaction, and opioid prescribing patterns, contributing to reductions in opioid-related deaths. Sharda et al. [61] demonstrated that billing data can identify complex cases, optimize resource utilization, and reduce readmissions and adverse drug events.

Financial data analytics and machine learning automate claims processing, reduce errors, and accelerate reimbursements [62,63]. Dey et al. [64] reported that AI models improve the detection of fraudulent claims by identifying anomalous behaviors in large-scale data. Business intelligence (BI) systems integrating financial, clinical, and administrative data improve decision-making, increase operational efficiency, and support patient safety [65].

King Saud Medical City’s implementation of an integrated revenue-cycle module reduced claim denial rates from 18% to 8% and decreased days in accounts receivable by 12 days within nine months [19]. A governance committee standardized documentation templates and sign-off procedures, reducing administrative staffing by 20% and enabling resource reallocation [10,19].

However, legacy clinical systems often lack the granularity required for accurate code mapping, and coders may distrust automated suggestions [9,11]. Data-quality issues and complex regulatory requirements further complicate design and validation, necessitating close collaboration among IT, clinical, finance, and compliance teams [9].

#### 3.2.7. Telemedicine Platforms

Telemedicine includes synchronous consultations through video or telephone, asynchronous store-and-forward services, and remote monitoring that may improve access, quality, and safety by enabling remote care and continuous monitoring. It has been particularly valuable for rural and underserved populations, as well as during the COVID-19 pandemic [35,66]. Telemonitoring can improve access to care by reducing travel requirements and facilitating follow-up, particularly for people who live in rural or underserved areas and for patients with chronic conditions and for palliative care [67]. Telemonitoring chronic conditions such as diabetes and hypertension improves control and reduces hospitalizations and emergency department visits [68,69]. It has been successfully applied in prenatal care and mental health [70], increasing treatment adherence and completion [71]. Evidence suggests that telemedicine can support chronic-disease monitoring and may reduce healthcare utilization in selected settings; however, outcomes depend on clinical indication, care model, connectivity, patient readiness, digital literacy, and integration with in-person services [38,68,69,71].

Patient safety benefits include reduced exposure to hospital-acquired infections and earlier detection of complications through remote monitoring [35,38]. However, disparities in internet access and digital literacy persist, particularly among low-income and older populations [68]. Targeted policies and support strategies are needed to ensure equitable access to telemedicine.

In Saudi Arabia, telemedicine adoption has been supported by national digital-health initiatives, but barriers remain, including variable digital literacy, technological access, provider acceptance, and evolving organizational and regulatory arrangements. Future studies should evaluate the quality, safety, equity, cost, and clinical outcomes of telemedicine across different Saudi healthcare settings.

#### 3.2.8. Artificial Intelligence and Retrieval-Augmented Generation in Healthcare

Artificial intelligence (AI) is increasingly embedded in healthcare to support diagnostic reasoning, treatment planning, risk prediction, medical imaging, triage, patient safety, clinical documentation, and health-service management [72,73]. Recent advances in machine learning, natural language processing (NLP), and large language models (LLMs) have expanded AI’s capacity to analyze large volumes of structured and unstructured health data, including EHRs, clinical notes, laboratory results, radiology reports, medical images, and biomedical literature [74,75]. Biomedical LLMs, including BioGPT and other domain-adapted transformer models, can support tasks such as medical question answering, information extraction, clinical-text summarization, and literature mining. Nevertheless, standalone LLMs have important limitations in high-stakes healthcare contexts: their knowledge may be outdated because it is encoded during training, their output may contain hallucinations or unsupported statements, and the basis for a generated response may be difficult for clinicians to verify. These limitations create risks for accuracy, traceability, patient privacy, equity, and clinical accountability [76].

Retrieval-augmented generation (RAG) is an AI architecture designed to address some of these limitations by combining an LLM with an external, searchable knowledge source. Rather than relying only on the model’s internal knowledge, a RAG system first retrieves relevant documents—such as clinical guidelines, peer-reviewed literature, institutional protocols, curated knowledge bases, or authorized EHR information—and then supplies this evidence to the generative model when producing a response [77]. The original RAG framework integrates a retriever and a generator, allowing answers to be conditioned on both the user’s question and retrieved documents. In healthcare, this approach may improve factual grounding, facilitate source traceability, and enable systems to incorporate updated medical knowledge without complete model retraining. Retrieval can be sparse, using lexical approaches such as TF–IDF or BM25, or dense, using semantic embeddings to identify conceptually similar clinical content despite variations in terminology, abbreviations, and phrasing [70].

Potential healthcare applications of RAG include diagnostic support, EHR and discharge summary generation, medical question answering, patient education, conversational agents, clinical trial matching, and biomedical literature synthesis. For example, RAG can retrieve a patient’s relevant medication history, laboratory trends, and previous admissions to support concise clinical summaries [78]. It can also retrieve specialty guidelines or peer-reviewed evidence to support clinician-facing decision assistance and patient-specific explanations. Studies reviewed by Neha et al. [70] indicate that RAG-based systems have been explored in nursing care, infectious diseases, liver disease, nephrology, thyroid disease, radiology, chronic pain, and ECG interpretation. However, most reported studies are early-stage technical evaluations, pilot applications, or benchmark-based assessments; therefore, apparent gains in factuality, relevance, or efficiency should not be interpreted as proof of improved patient outcomes without rigorous clinical validation.

Safe adoption requires more than technical performance. Healthcare RAG systems must retrieve information from high-quality, current, and appropriately governed sources; protect confidential patient data through access controls, de-identification, audit trails, and secure indexing; and provide clear links between generated statements and their supporting evidence [79]. Key implementation challenges include retrieval noise, domain shift across hospitals and documentation systems, latency, incomplete or unstructured clinical data, limited explainability, algorithmic bias, knowledge drift, and insufficient integration with clinical workflows [70]. Evaluation should therefore extend beyond general NLP metrics such as BLEU or ROUGE to include retrieval relevance, factual consistency, clinical validity, source traceability, potential harm, and expert review. RAG should be implemented as clinician-support technology—not as an autonomous replacement for professional judgment—with human oversight, continuous monitoring, and periodic updating of the underlying evidence base.

### 3.3. Improving Quality and Safety in Healthcare Services

DT reshapes workflows and organizational models, enabling a shift from reactive problem-solving to proactive quality assurance [9,13]. Quality improvement efforts use EHR-based dashboards, CDS, RPA, and patient portals to monitor performance, standardize care, and extend quality efforts beyond hospital walls [19].

In Saudi hospitals, integrated digital dashboards have increased compliance with clinical guidelines by 18% and reduced hospital-acquired infections by 12% [7]. Analytics dashboards aggregate near-real-time data on key performance indicators and enable timely interventions for outliers [13]. CDS systems embed guideline-based recommendations into clinicians’ workflows, increasing adherence to best-practice bundles and improving care consistency [9]. RPA reduces errors and turnaround times in laboratory processes, improving the accuracy and reliability of diagnostic information [13].

Patient portals and mobile applications extend quality initiatives beyond healthcare facilities by providing access to care plans, educational materials, and discharge instructions. Improved engagement and understanding of discharge information have been associated with reduced readmissions among patients with chronic conditions [19].

In a 500-bed tertiary care center in Riyadh, the completeness of Computerized Provider Order Entry (CPOE) data improved significantly over time, increasing from 5.5% in 2015 to 26.0% in 2017 and 49.5% in 2019. Multivariable analysis identified year of documentation as the strongest predictor of completeness (OR = 17.47 for 2019 vs. 2015, *p* < 0.0001), with length of hospitalization also showing a modest association (OR = 1.04, *p* = 0.045). Pharmacist-led medication reconciliation was associated with higher completeness in bivariate analysis (2.5-fold increase, *p* < 0.0001). Despite these gains, gaps persisted in key clinical variables [8].

Safety-focused digital interventions include CPOE, BCMA, automated incident reporting, predictive safety dashboards, and Internet of Things (IoT) sensors [13]. CPOE eliminates illegible handwriting and enforces standardized orders, while BCMA verifies the “five rights” of medication administration at the bedside [13]. Automated incident-reporting systems and safety dashboards improve detection of near misses and adverse events. IoT sensors and wearables support early detection of patient deterioration and reduce falls and unplanned intensive care transfers [13].

Medication-error tracking is a key safety indicator enhanced by digital tools. At the bedside, barcode scanning reduced dispensing errors by 30%, while automated alerts for potential drug–allergy interactions prevented 45 adverse drug-event risks per 1000 patients [13]. Dal Mas et al. [9] emphasized that real-time event logging and integrated incident-reporting platforms support proactive safety cultures by enabling teams to address near misses before harm occurs.

Digital platforms also strengthen patient engagement and perceived safety. Frisinger and Papachristou [10] reported a mean satisfaction score of 4.7/5 among telemedicine users, with 88% of patients indicating increased confidence in their care plans. In the study by Vărzaru and Bocean [19], patient access to personalized EHR portals was associated with a 20% increase in self-reported understanding of treatment instructions, which was linked to fewer readmissions and higher adherence rates.

Economic analyses show that upfront technology investments can generate substantial downstream savings. Vărzaru and Bocean [19] estimated an average return on investment (ROI) of 2.3 over three years following the implementation of an end-to-end digital records and analytics platform. Dal Mas et al. [9] reported that every dollar invested in interoperability and data-governance infrastructure can yield up to three dollars in operational efficiency through reduced administrative overhead and fewer preventable adverse events. Together, these findings underscore the need to view DT as a strategic approach to enhancing quality, safety, patient experience, and organizational sustainability.

Alert fatigue and workforce resistance remain important barriers, with override rates exceeding 70% when alerts are non-actionable [9,11]. Co-design approaches, targeted training, multidisciplinary governance, and iterative alert tuning are therefore essential to align digital quality and safety initiatives with clinical priorities and national health-sector strategies [9,10].

### 3.4. Challenges of Digital Transformation in Saudi Healthcare

Despite policy support through Vision 2030, digital transformation in Saudi healthcare faces several interconnected challenges. These include variations in digital infrastructure and interoperability, uneven implementation of advanced electronic safety functions, workforce training needs, resistance to workflow changes, and data governance and cybersecurity requirements [20,80]. For example, pharmacy information system functionality and adoption have varied across hospitals in the Eastern Province, with limited implementation of barcode-assisted medication administration and electronic adverse event monitoring reported in some settings. Addressing these challenges requires coordinated governance, interoperable standards, sustained investment in digital infrastructure, continuous staff development, and active clinical engagement during implementation.

#### 3.4.1. Infrastructure and Interoperability Challenges

Fragmented IT environments and a lack of a unified national infrastructure characterize Saudi Arabia’s digital health landscape. Many hospitals rely on legacy systems with proprietary data structures and outdated interfaces, hindering seamless data exchange and analytics aggregation [43]. In three hospitals in the Eastern Province of Saudi Arabia, pharmacy information systems were integrated with electronic health records and supported most prescribing and transcription functions; however, drug dispensing remained largely manual. Barcode-assisted medication administration and electronic adverse drug event monitoring were not implemented. Overall utilization of system functionalities was low, with the highest use in patient administration records (56.8%) and the lowest in pharmacy stock linkage (9.1%), highlighting the need to improve user engagement [17]. Addressing these gaps requires national mandates for data standards such as HL7 FHIR, investment in shared health information exchanges, and incentives for vendors to adopt open APIs [9,12].

#### 3.4.2. Workforce Resistance and Cultural Barriers

DT initiatives frequently encounter clinician skepticism and workaround behaviors driven by perceived threats to professional autonomy and increased documentation burden [11]. Hierarchical decision-making structures can limit frontline involvement in design and reduce user ownership [10]. In Saudi RPA and telemedicine pilots, utilization initially remained low until digital champions were deployed to mentor staff, after which adoption increased substantially [15]. Structured change-management strategies, continuous training, co-design approaches, and visible leadership commitment are therefore essential [9,10].

#### 3.4.3. Governance, Policy, and Regulatory Constraints

Digital health governance in Saudi Arabia is fragmented across the Ministry of Health, the Saudi Food and Drug Authority (SFDA), and hospital governing boards, complicating coordination [81]. Overlapping procurement, cybersecurity, and data-governance requirements create regulatory ambiguity and delay project certification [9]. The absence of clear guidance on medical software and AI tools within software-as-a-medical-device frameworks has led institutions to adopt cautious, wait-and-see approaches despite promising pilot results [13].

Vision 2030’s digital-health agenda has prompted revisions to consent, privacy, and data-sharing regulations. For example, early cross-hospital EHR exchange pilots failed due to non-standardized consent forms, prompting the development of a universal consent model that enabled interoperable information exchange [20]. Transparent, integrated governance and policy frameworks are necessary to scale digital initiatives nationwide [9].

#### 3.4.4. Funding, Economics, and Sustainability Challenges

DT requires substantial capital investment, whereas financial returns often emerge over extended periods [9]. Budget silos separating capital IT expenditures from downstream operational savings complicate reinvestment in maintenance and training [12]. Emerging funding mechanisms, such as public–private partnerships and performance-based risk-sharing contracts, have been tested in national EHR pilots, distributing financial risk while incentivizing measurable improvements [19].

#### 3.4.5. Digital Literacy and Training Gaps

One-time go-live training is inadequate for sustained, safe use of digital tools [11]. Without continuous, role-specific education and support, staff often revert to manual workflows, leading to underutilization and increased error risk [11]. During EMR and telemedicine implementations in Saudi hospitals, documentation errors and aborted virtual visits increased when training and on-demand support were insufficient, but declined after targeted refresher webinars and on-site coaching [14]. Institutions are increasingly institutionalizing continuous learning programs and embedding digital competencies into performance appraisals [10].

#### 3.4.6. Technological Resilience and Management of Change

Technological resilience is essential to prevent service disruptions caused by system outages, cyberattacks, or software failures. Tortorella et al. [13] emphasized disaster-recovery preparedness and redundant architecture, alongside cybersecurity measures such as network segmentation and intrusion detection. Alert fatigue also threatens safety; override rates exceed 70% when alerts are perceived as non-actionable, undermining trust in CDS [9]. Iterative alert tuning, phased rollouts, and structured feedback processes help align alerts with clinical priorities and maintain user engagement [9,10].

An evidence-characterization table was added as Supplementary Data (Table S2).

## 4. Discussion

Saudi Arabia has prioritized digital health transformation as part of Vision 2030, with EHRs, CPOE, digital prescribing, telemedicine, and other health information systems expected to support quality, safety, access, and operational efficiency [82]. EHRs, EMRs, computerized provider order entry, digital prescribing, telemedicine, online appointment systems, clinical and pharmacy automation, health-data analytics, and emerging AI-enabled tools can serve complementary functions. However, the available evidence is heterogeneous and largely derived from international or single-institution settings; therefore, reported benefits should be interpreted as potentially relevant implementation lessons rather than as definitive evidence of effectiveness across Saudi healthcare organizations.

Evidence from Saudi settings indicates that electronic documentation and prescribing systems can improve the completeness and safety of clinical processes, but the benefits depend on system maturity, infrastructure, workflow integration, and user engagement. Persistent gaps in the use of advanced safety functions, including barcode-assisted medication administration and electronic adverse-event monitoring, may limit the full realization of patient-safety benefits in some institutions [7].

Saudi evidence indicates that documentation completeness and electronic prescribing performance can improve as systems mature and as infrastructure, workflow integration, training, pharmacist involvement, and user engagement are strengthened. These systems can also enable clinical decision support, medication reconciliation, audit processes, and population-level quality monitoring [8]. Nevertheless, incomplete data entry, fragmented systems, limited interoperability, alert fatigue, and uneven uptake of advanced safety functions may restrict their contribution to patient safety. Accordingly, Saudi implementation strategies should prioritize interoperable information exchange, high-quality documentation, clinically relevant decision support, and continuous monitoring of medication-related and documentation-related outcomes.

Telemedicine, remote monitoring, patient portals, and online appointment systems play different but equally important roles: they can extend access beyond the hospital, support continuity of care, reduce travel burden, facilitate follow-up, and improve patient engagement. These technologies may be particularly relevant for patients with chronic conditions, people living in remote areas, and individuals who face difficulty attending in-person appointments [35]. At the same time, their value depends on connectivity, affordability, language accessibility, privacy, digital literacy, clinical appropriateness, and integration with in-person services. To prevent digital transformation from widening inequities, Saudi health services should assess who uses these services, who is excluded, and whether remote models achieve comparable safety, access, satisfaction, and continuity outcomes across rural, older, lower-literacy, and clinically vulnerable populations.

Clinical and administrative automation, data analytics, claims systems, and AI-enabled tools may support efficiency, quality improvement, and resource management. Automation may reduce repetitive workloads in laboratories, pharmacies, scheduling, documentation, and administrative processes, whereas dashboards and analytics can help organizations identify performance variations, monitor key indicators, and allocate resources more effectively [83]. Claims and financial data systems can complement clinical data by providing information on utilization, reimbursement, readmissions, and service patterns. However, efficiency gains should not automatically be interpreted as improvements in quality or safety. Saudi implementation should evaluate automation and analytics using patient-centered, workforce, financial, and safety measures, including turnaround time, clinician workload, data quality, error rates, patient experience, resource use, and cost-effectiveness.

AI-based clinical decision support, predictive analytics, and generative-AI tools represent a developing area of digital transformation rather than a replacement for clinical judgment [84]. Their potential value lies in supporting risk stratification, early warning, documentation, triage, and evidence retrieval; however, their implementation raises additional concerns regarding algorithmic bias, data representativeness, explainability, validation, cybersecurity, privacy, accountability, and automation bias [85]. Before broad deployment in Saudi healthcare, AI-enabled tools require local validation against clinically meaningful outcomes, transparent governance, clinician-in-the-loop oversight, and mechanisms for auditing, monitoring, and correcting unsafe or inequitable outputs.

Evidence from Saudi hospitals and international studies indicates that successful digital transformation depends not only on system availability but also on robust infrastructure, structured training, effective change management, and continuous workflow optimization [43]. In line with this, the current literature review revealed that infrastructure sufficiency and positive staff attitudes are important drivers of better use of electronic systems. At the same time, limited adoption of advanced safety functionalities (such as barcode medication administration and automated adverse event monitoring) constrains the full realization of Vision 2030 objectives for patient safety [16]. The observed association between pharmacist-led medication reconciliation and greater data completeness further underscores the value of multidisciplinary collaboration and supports national policies that promote clinical pharmacy services as part of digital safety strategies.

The relative importance of these technologies lies in their integration. EHRs and interoperable data infrastructure provide the core information layer; e-prescribing, pharmacy automation, and decision support strengthen medication safety; telemedicine, portals, and appointment systems improve access and patient engagement; automation and analytics support operational performance; and AI-enabled tools may add value when grounded in reliable data and strong governance. The success of Saudi healthcare transformation will therefore depend less on the number of digital tools adopted than on whether they are interoperable, clinically usable, equitably accessible, securely governed, supported by workforce capability, and evaluated against meaningful quality, safety, access, and economic outcomes.

Strength of the evidence: The evidence synthesized in this review varies substantially in design, methodological rigor, and relevance to Saudi healthcare. Much of the evidence on EHRs, electronic prescribing, pharmacy automation, appointment systems, telemedicine, and administrative automation is derived from observational studies, retrospective before-and-after evaluations, service audits, pilot initiatives, qualitative studies, or secondary reports. Although systematic reviews and meta-analyses are available for some interventions, particularly electronic prescribing and electronic medication-management strategies, randomized controlled trials and robust comparative evaluations remain limited.

Accordingly, reported reductions in medication errors, improvements in patient satisfaction, operational efficiencies, cost savings, and return on investment should not be interpreted as uniform causal effects of digital transformation. Outcomes may reflect differences in baseline performance, intervention design, implementation fidelity, organizational readiness, concurrent quality-improvement activities, staff training, follow-up duration, and outcome definitions. The Saudi-specific evidence base remains relatively limited and is predominantly descriptive or observational. Therefore, the findings of this narrative review indicate potential benefits and implementation considerations rather than definitive estimates of effectiveness or economic value. Saudi healthcare organizations also vary considerably in digital maturity and access to specialized technical resources. Accordingly, national policy and institutional decisions should be informed by local implementation and outcome evaluations rather than by extrapolation from international effect estimates alone.

From a policy perspective, these findings support the need for national and institutional measures that go beyond technical implementation, including mandating “hard stops” for core data elements, ensuring dedicated training and support for clinicians, and embedding continuous audit and feedback mechanisms to monitor data quality and system use [86]. Strengthening governance around digital documentation, fostering collaboration among IT teams, clinical staff, and hospital leadership, and aligning local implementation plans with Vision 2030’s emphasis on quality, efficiency, and patient-centered care will be essential to translate digital infrastructure into measurable improvements in outcomes [87]. Future work in Saudi Arabia should examine how different hospital types and regions adopt and sustain these practices and evaluate the impact of advanced digital safety tools on clinical outcomes to guide the next phase of the national digital health transformation.

### Limitations

This narrative review has several limitations that should be considered when interpreting its findings. First, although a structured search strategy, predefined eligibility criteria, and transparent screening procedures were used, the narrative review design did not include a formal risk-of-bias assessment, a certainty-of-evidence rating, or a quantitative meta-analysis. Therefore, the review cannot establish the magnitude of the effect of individual digital interventions or infer causal relationships between digital transformation and healthcare quality or patient safety outcomes.

Second, the included evidence was heterogeneous with respect to study design, healthcare setting, patient population, digital intervention, implementation maturity, comparator, and outcome measures. The reviewed technologies ranged from EHRs and computerized provider order entry to telemedicine, automation, pharmacy dispensing systems, online appointment platforms, and data analytics. This heterogeneity limited direct comparisons across studies and precluded pooled quantitative analysis.

Third, much of the available evidence originated from high-income settings outside Saudi Arabia. Consequently, the findings may not be fully transferable to Saudi healthcare organizations, which differ in infrastructure, interoperability, workforce characteristics, governance arrangements, financing mechanisms, regulatory requirements, and digital health maturity. Saudi-specific research was also unevenly distributed across technologies, with comparatively limited empirical evidence for some interventions and care settings.

Fourth, the review included only English- and Arabic-language sources and relied in part on Google Scholar, citation tracking, and relevant grey literature. In addition, the rapidly evolving nature of digital health means that newer technologies, regulations, and implementation outcomes may emerge after the March 2026 search end date.

Despite these limitations, the structured, broad search strategy facilitated the integration of empirical evidence, conceptual frameworks, and policy documents relevant to Saudi Arabia. The findings should be interpreted as a context-sensitive synthesis intended to identify major patterns, implementation considerations, and research priorities, rather than as pooled estimates of intervention effectiveness.

## 5. Conclusions

Within the limitations of a narrative synthesis and the heterogeneous evidence base, this review indicates that DT may strengthen healthcare quality, patient safety, access, and operational efficiency through EHRs and CPOE, electronic prescribing, online appointment systems, telemedicine, clinical and pharmacy automation, data analytics, and digital clinical decision-support tools. However, these potential benefits are not automatic consequences of technology adoption. They depend on interoperability, high-quality data, workflow integration, user-centered design, workforce competence, clinical leadership, robust governance, cybersecurity safeguards, sustainable financing, and continuous monitoring of unintended consequences.

Importantly, a substantial proportion of the available evidence comes from technologically advanced health systems outside Saudi Arabia. Findings from these settings should therefore be interpreted as informative but not directly transferable to Saudi healthcare organizations. Differences in health-system structure, public–private service delivery, digital maturity, health-information infrastructure, workforce composition, patient digital literacy, financing arrangements, regulatory requirements, and data-governance practices may influence implementation processes and outcomes. Saudi-specific evidence remains limited, unevenly distributed across technologies and regions, and often based on single-institution, pilot, cross-sectional, retrospective, or descriptive studies. Consequently, claims regarding clinical effectiveness, safety, equity, cost savings, and return on investment require locally generated evidence.

Future research should move beyond general descriptions of digital adoption and undertake coordinated, multicenter, longitudinal, and mixed-methods implementation studies across primary care, hospitals, public and private providers, and urban and rural regions of Saudi Arabia. Quantitative evaluations should examine national and cross-institutional EHR interoperability; data completeness and exchange; medication-prescribing, dispensing, and administration errors; adverse drug events; clinical-decision-support alert performance and alert fatigue; waiting times; missed appointments; emergency-department use; hospital readmissions; clinical outcomes; workflow efficiency; clinician workload; and patient-reported experience and access measures. Studies should also evaluate digital literacy among patients and healthcare professionals, with particular attention to older adults, persons with chronic illness, rural populations, and other groups at risk of digital exclusion.

Priority areas include evaluating the safety, effectiveness, usability, and equity implications of telemedicine; assessing AI-based clinical decision-support systems for diagnostic accuracy, clinical utility, bias, transparency, and human oversight; measuring national EHR interoperability using standardized data-exchange indicators; and conducting cybersecurity and data-governance readiness assessments that examine access control, consent, privacy, incident response, system resilience, and continuity-of-care planning. Economic studies should use long-term, transparent cost-effectiveness approaches that account for technology acquisition, maintenance, interoperability, cybersecurity, training, workflow redesign, implementation support, healthcare utilization, and patient costs, rather than relying only on short-term operational savings or estimated return on investment.

Qualitative components should explore the experiences of clinicians, patients, managers, information-technology teams, and policymakers, including perceived usefulness, usability, trust, workload, training needs, workflow disruption, privacy concerns, and organizational readiness. Integrating these findings with quantitative outcome measures will help determine not only whether a digital intervention works, but also whom it works for, under which conditions, at what cost, and how it can be sustainably scaled. Such context-sensitive evidence is essential for guiding the equitable and safe implementation of Vision 2030 digital-health priorities and for translating digital infrastructure into measurable improvements in healthcare quality and patient safety.

## Data Availability

The original contributions presented in this study are included in the article/Supplementary Materials. Further inquiries can be directed to the corresponding author.

## Supplementary Materials

The following supporting information can be downloaded at: https://www.mdpi.com/article/10.3390/healthcare14172810/s1. Table S1. Characteristics of empirical studies included in the narrative review [4,5,7,8,14,15,16,17,21,22,23,24,25,26,27,28,29,30,31,32,33,34,35,36,37,38,39,42,43,44,45,46,47,48,49,50,51,52,53,54,55,56,57,58,59,60,61,62,63,64,65,66,67,68,69,70,71,72,73,74,75,76,77,78]; Table S2. Characteristics and strength of evidence on digital transformation interventions and outcomes.

## References

[1] Vial G. (2019). Understanding digital transformation: A review and a research agenda. J. Strateg. Inf. Syst..

[2] Stoumpos A.I., Kitsios F., Talias M.A. (2023). Digital Transformation in Healthcare: Technology Acceptance and Its Applications. Int. J. Environ. Res. Public Health.

[3] Busse R., Panteli D., Quentin W. (2019). Improving Healthcare Quality in Europe: Characteristics, Effectiveness and Implementation of Different Strategies.

[4] Alajwari H.A., Alfayez A., Alsalman D., Alanezi F., Alhodaib H., Al-Rayes S., Aljaffary A., AlThani B., AlNujaidi H., Al-Saif A.K. (2022). Knowledge and attitude of Saudi Arabian citizens towards telemedicine during the COVID-19 pandemic. Int. Health.

[5] Almalki A., Jambi A., Elbehiry B., Albuti H. (2023). Improving Inpatient Medication Dispensing with an Automated System. Glob. J. Qual. Saf. Healthc..

[6] Jagadamba G., Shashidhar R., Ravi V., Mallu S., Alahmadi T.J. (2024). Design of Interoperable Electronic Health Record (EHR) Application for Early Detection of Lung Diseases Using a Decision Support System by Expanding Deep Learning Techniques. Open Respir. Med. J..

[7] Al-Kahtani N., Alruwaie S., Al-Zahrani B.M., Abumadini R.A., Aljaafary A., Hariri B., Alissa K., Alakrawi Z., Alumran A. (2022). Digital health transformation in Saudi Arabia: A cross-sectional analysis using Healthcare Information and Management Systems Society’ digital health indicators. Digit. Health.

[8] Alanazi H.S., Alruthia Y. (2026). Evolution of Computerized Provider Order Entry Documentation at a Leading Tertiary Care Referral Center in Riyadh. Healthcare.

[9] Dal Mas F., Massaro M., Rippa P., Secundo G. (2023). The challenges of digital transformation in healthcare: An interdisciplinary literature review, framework, and future research agenda. Technovation.

[10] Frisinger A., Papachristou P. (2024). Bridging the voice of healthcare to digital transformation in practice—A holistic approach. BMC Digit. Health.

[11] Kakale M.M. (2024). Of digital transformation in the healthcare (systematic review of the current state of the literature). Health Technol..

[12] Kraus S., Durst S., Ferreira J.J., Veiga P., Kailer N., Weinmann A. (2022). Digital transformation in business and management research: An overview of the current status quo. Int. J. Inf. Manag..

[13] Tortorella G.L., Narayanamurthy G., Thurer M. (2021). Identifying pathways to a high-performing lean automation implementation: An empirical study in the manufacturing industry. Int. J. Prod. Econ..

[14] Alhur A. (2024). Overcoming Electronic Medical Records Adoption Challenges in Saudi Arabia. Cureus.

[15] Alodhialah A.M., Almutairi A.A., Almutairi M. (2024). Telehealth Adoption Among Saudi Older Adults: A Qualitative Analysis of Utilization and Barriers. Healthcare.

[16] Alshamrani M.M., Alzahrani A.R., Alqahtani A.F.A., Almutairi B.H., Aloraini A.M., Al-Balawi H.A.S., Alshammari M.A.R., Alsloubi M.F.H., Alshammary W.M. (2025). Adoption and Effectiveness of Automated Dispensing Cabinets in Reducing Medication Errors in Saudi Tertiary Care Centers. Rev. Diabet. Stud..

[17] El Mahalli A., El-Khafif S.H., Yamani W. (2016). Assessment of Pharmacy Information System Performance in Three Hospitals in Eastern Province, Saudi Arabia. Perspect. Health Inf. Manag..

[18] Ghanad A. (2023). An Overview of Quantitative Research Methods. Int. J. Multidiscip. Res. Anal..

[19] Vărzaru A.A., Bocean C.G. (2025). Systemic interactions among digital transformation, sustainable orientation, and economic outcomes in EU countries. Systems.

[20] Mani Z.A., Goniewicz K. (2024). Transforming healthcare in Saudi Arabia: A comprehensive evaluation of vision 2030’s impact. Sustainability.

[21] Atasoy H., Greenwood B.N., McCullough J.S. (2019). The Digitization of Patient Care: A Review of the Effects of Electronic Health Records on Health Care Quality and Utilization. Annu. Rev. Public Health.

[22] Ayaad O., Alloubani A., Abu Alhajaa E., Farhan M., Abuseif S., Al Hroub A., Akhu-Zaheya L. (2019). The role of electronic medical records in improving the quality of health care services: Comparative study. Int. J. Med. Inform..

[23] Gatiti P., Ndirangu E., Mwangi J., Mwanzu A., Ramadhani T. (2021). Enhancing healthcare quality in hospitals through electronic health records: A systematic review. J. Health Inform. Dev. Ctries..

[24] Upadhyay S., Hu H.F. (2022). A Qualitative Analysis of the Impact of Electronic Health Records (EHR) on Healthcare Quality and Safety: Clinicians’ Lived Experiences. Health Serv. Insights.

[25] Uslu A., Stausberg J. (2021). Value of the Electronic Medical Record for Hospital Care: Update From the Literature. J. Med. Internet Res..

[26] Wani D., Malhotra M.K. (2018). Does the meaningful use of electronic health records improve patient outcomes. J. Oper. Manag..

[27] Alanazi W.K., Almutairi S.H., Alamri A.A., Alsubaie M.F., Tohary O.M., Hussein M.T., Alshamrani M.H., Alharbi G.G., Alsomali H.M., Alsadran Q.T. (2024). Effect of electronic prescription system modifications on reducing prescribing errors in a military hospital. J. Pharm. Policy Pract..

[28] Alzahrani T., Binsaad J., Almasabi A., Alharthi F., Alqarni A., Albaqami S., Alqahtani M., Aljabry I. (2024). The impact of electronic prescribing systems on clinical pharmacy practice. Int. J. Community Med. Public Health.

[29] Camacho E.M., Gavan S., Keers R.N., Chuter A., Elliott R.A. (2024). Estimating the impact on patient safety of enabling the digital transfer of patients’ prescription information in the English NHS. BMJ Qual. Saf..

[30] Cassidy C.E., Boulos L., McConnell E., Barber B., Delahunty-Pike A., Bishop A., Fatima N., Higgins A., Churchill M., Lively A. (2023). E-prescribing and medication safety in community settings: A rapid scoping review. Explor. Res. Clin. Soc. Pharm..

[31] Porterfield A., Engelbert K., Coustasse A. (2014). Electronic prescribing: Improving the efficiency and accuracy of prescribing in the ambulatory care setting. Perspect. Health Inf. Manag..

[32] Roumeliotis N., Sniderman J., Adams-Webber T., Addo N., Anand V., Rochon P., Taddio A., Parshuram C. (2019). Effect of Electronic Prescribing Strategies on Medication Error and Harm in Hospital: A Systematic Review and Meta-analysis. J. Gen. Intern. Med..

[33] Rozenblum R., Rodriguez-Monguio R., Volk L.A., Forsythe K.J., Myers S., McGurrin M., Williams D.H., Bates D.W., Schiff G., Seoane-Vazquez E. (2020). Using a Machine Learning System to Identify and Prevent Medication Prescribing Errors: A Clinical and Cost Analysis Evaluation. Jt. Comm. J. Qual. Patient Saf..

[34] Shaikh S., Furniss D., Blandford A., McLeod M., Ma T., Beykloo M.Y., Franklin B.D. (2019). The impact of electronic prescribing systems on healthcare professionals’ working practices in the hospital setting: A systematic review and narrative synthesis. BMC Health Serv. Res..

[35] Ezeamii V.C., Okobi O.E., Wambai-Sani H., Perera G.S., Zaynieva S., Okonkwo C.C., Ohaiba M.M., William-Enemali P.C., Obodo O.R., Obiefuna N.G. (2024). Revolutionizing Healthcare: How Telemedicine Is Improving Patient Outcomes and Expanding Access to Care. Cureus.

[36] Iftikhar L., Iftikhar M.F., Hanif M.I. (2023). Docgpt: Impact of chatgpt-3 on health services as a virtual doctor. EC Paediatr..

[37] Kammrath Betancor P., Boehringer D., Jordan J., Lüchtenberg C., Lambeck M., Ketterer M.C., Reinhard T., Reich M. (2025). Efficient patient care in the digital age: Impact of online appointment scheduling in a medical practice and a university hospital on the “no-show”-rate. Front. Digit. Health.

[38] Khoong E.C., Sharma A.E., Gupta K., Adler-Milstein J., Sarkar U. (2022). The Abrupt Expansion of Ambulatory Telemedicine: Implications for Patient Safety. J. Gen. Intern. Med..

[39] Zanaboni P., Fagerlund A.J. (2020). Patients’ use and experiences with e-consultation and other digital health services with their general practitioner in Norway: Results from an online survey. BMJ Open.

[40] Din S.M.S.U. (2025). Global Digital Transformation in Healthcare: Impact of Connected Healthcare Systems on Patient Safety. Glob. J. Qual. Saf. Healthc..

[41] Adekunle Oyeyemi A., Jeremiah Olawumi A., Chioma Anthonia O., Rawlings C., Oloruntoba B. (2024). Ethical considerations in healthcare IT: A review of data privacy and patient consent issues. World J. Adv. Res. Rev..

[42] Alghamdi A.A., Sayed S.H. (2026). Challenges of Electronic Medical Record Implementation From the Perspective of Hospital Leaders in Riyadh, Kingdom of Saudi Arabia’s Public Hospitals: A Mixed-Methods Study. Saudi Med. J..

[43] Alshamrani N.S. (2025). Digital Health Transformation Through EHRs in Saudi Arabia: Operational Insights and MIS Implications. Int. J. E-Serv. Mob. Appl. (IJESMA).

[44] Shen X., Yang W., Sun S. (2019). Analysis of the Impact of China’s Hierarchical Medical System and Online Appointment Diagnosis System on the Sustainable Development of Public Health: A Case Study of Shanghai. Sustainability.

[45] Carini E., Villani L., Pezzullo A.M., Gentili A., Barbara A., Ricciardi W., Boccia S. (2021). The Impact of Digital Patient Portals on Health Outcomes, System Efficiency, and Patient Attitudes: Updated Systematic Literature Review. J. Med. Internet Res..

[46] Ng’andu D., Haabazoka L. (2024). A study of the effect of health records digitalization on healthcare facility operational efficiency. Open J. Bus. Manag..

[47] Dendere R., Slade C., Burton-Jones A., Sullivan C., Staib A., Janda M. (2019). Patient Portals Facilitating Engagement With Inpatient Electronic Medical Records: A Systematic Review. J. Med. Internet Res..

[48] Huang N., Yan Z., Yin H. (2021). Effects of online–offline service integration on e-healthcare providers: A quasi-natural experiment. Prod. Oper. Manag..

[49] Al Naam Y.A., Elsafi S., Al Jahdali M.H., Al Shaman R.S., Al-Qurouni B.H., Al Zahrani E.M. (2022). The Impact of Total Automaton on the Clinical Laboratory Workforce: A Case Study. J. Healthc. Leadersh..

[50] Jones S., Thompson K., Porter B., Shepherd M., Sapkaroski D., Grimshaw A., Hargrave C. (2024). Automation and artificial intelligence in radiation therapy treatment planning. J. Med. Radiat. Sci..

[51] Sharaf N.I. (2024). Prescription Automation: Enhancing Medication Safety and Healthcare Efficiency. Gland. Surg..

[52] Meknassi Salime G., Bhirich N., Cherif Chefchaouni A., El Hamdaoui O., El Baraka S., Elalaoui Y. (2025). Assessment of Automation Models in Hospital Pharmacy: Systematic Review of Technologies, Practices, and Clinical Impacts. Hosp. Pharm..

[53] Huang W.L., Liao S.L., Huang H.L., Su Y.X., Jerng J.S., Lu C.Y., Ho W.S., Xu J.R. (2024). A case study of lean digital transformation through robotic process automation in healthcare. Sci. Rep..

[54] Chakhunashvili A., Blommengren A., Kullberg A. (2025). Implementation of Automated PREM Process to Better Capture Patients’ Overall Experience of Care Services at Karolinska University Hospital. Int. J. Health Plan. Manag..

[55] Tu H.N., Shan T.H., Wu Y.C., Shen P.H., Wu T.Y., Lin W.L., Yang-Kao Y.H., Cheng C.L. (2023). Reducing Medication Errors by Adopting Automatic Dispensing Cabinets in Critical Care Units. J. Med. Syst..

[56] Alanazi M.F., Shahein M.I., Alsharif H.M., Alotaibi S.M., Alanazi A.O., Alanazi A.O., Alharbe U.A., Almfalh H.S.S., Amirthalingam P., Hamdan A.M. (2022). Impact of automated drug dispensing system on patient safety. Pharm. Pract..

[57] Boyd A., Chaffee B. (2019). Critical evaluation of pharmacy automation and robotic systems: A call to action. Hosp. Pharm..

[58] Zheng W.Y., Lichtner V., Van Dort B.A., Baysari M.T. (2021). The impact of introducing automated dispensing cabinets, barcode medication administration, and closed-loop electronic medication management systems on work processes and safety of controlled medications in hospitals: A systematic review. Res. Soc. Adm. Pharm..

[59] Craswell A., Bennett K., Hanson J., Dalgliesh B., Wallis M. (2021). Implementation of distributed automated medication dispensing units in a new hospital: Nursing and pharmacy experience. J. Clin. Nurs..

[60] Kaiser V., Fichtner U.A., Schmuker C., Gunster C., Rau D., Staab L., Farin-Glattacker E. (2023). A cross-sectoral approach to utilizing health claims data for quality assurance in medical rehabilitation: Study protocol of a combined prospective longitudinal and retrospective cohort study. BMC Health Serv. Res..

[61] Sharda M., Sharma S., Raikar S., Verhagen N., Wagle J., Mathur R., Gowda S., Kommu S., Prasad R., Bhandari S. (2025). The Role of Machine Learning in Predicting Hospital Readmissions Among General Internal Medicine Patients: A Systematic Review. Cureus.

[62] Erickson S.M., Outland B., Joy S., Rockwern B., Serchen J., Mire R.D., Goldman J.M., Medical P., Quality Committee of the American College of Physicians (2020). Envisioning a Better U.S. Health Care System for All: Health Care Delivery and Payment System Reforms. Ann. Intern. Med..

[63] Zouo S.J.C., Olamijuwon J. (2024). Financial data analytics in healthcare: A review of approaches to improve efficiency and reduce costs. Open Access Res. J. Sci. Technol..

[64] Dey R., Roy A., Akter J., Mishra A., Sarkar M. (2025). AI-driven machine learning for fraud detection and risk management in U.S. healthcare billing and insurance. J. Comput. Sci. Technol. Stud..

[65] Nwosu N.T. (2024). Reducing operational costs in healthcare through advanced BI tools and data integration. World J. Adv. Res. Rev..

[66] Palozzi G., Schettini I., Chirico A. (2020). Enhancing the sustainable goal of access to healthcare: Findings from a literature review on telemedicine employment in rural areas. Sustainability.

[67] Ibrahim A.M., Sweelam R.K., Hassabelnaby F.G.E., Mohamed Abu Negm L.M., Zaghamir D.E.F., Elneblawi N.H., Ahmed S.I., Mohamed L.Z.G., Shahin M.A.H. (2026). Nurse-led tele-palliative care for symptom management and family support: A hybrid umbrella review of reviews and primary studies. Palliat. Support. Care.

[68] Baughman D.J., Jabbarpour Y., Westfall J.M., Jetty A., Zain A., Baughman K., Pollak B., Waheed A. (2022). Comparison of Quality Performance Measures for Patients Receiving In-Person vs Telemedicine Primary Care in a Large Integrated Health System. JAMA Netw. Open.

[69] Fieux M., Duret S., Bawazeer N., Denoix L., Zaouche S., Tringali S. (2020). Telemedicine for ENT: Effect on quality of care during COVID-19 pandemic. Eur. Ann. Otorhinolaryngol. Head. Neck Dis..

[70] Neha F., Bhati D., Shukla D.K. (2025). Retrieval-Augmented Generation (RAG) in Healthcare: A Comprehensive Review. AI.

[71] Society for Maternal-Fetal M., Patient S., Quality C., Healy A., Davidson C., Allbert J., Bauer S., Toner L., Combs C.A. (2023). Society for Maternal-Fetal Medicine Special Statement: Telemedicine in obstetrics—quality and safety considerations. Am. J. Obstet. Gynecol..

[72] Fahim Y.A., Hasani I.W., Kabba S., Ragab W.M. (2025). Artificial intelligence in healthcare and medicine: Clinical applications, therapeutic advances, and future perspectives. Eur. J. Med. Res..

[73] Mariano M.E.M., Shahin M.A.H., Ancheta S.J., Kunjan M.V., Al Dossary N.M., Al Ojaimi S.F., Al Qudah S.A., Al Harbi H.F. (2025). Exploring artificial intelligence knowledge, attitudes, and practices among nurses, faculty, and students in Saudi Arabia: A cross-sectional analysis. Soc. Sci. Humanit. Open.

[74] Omer A., Alkhir M.A., Yousef M., Shahin M.A.H., Osman H.E., Manssor E.H., Zidan M.M.A., Abbas W., Hassan K. (2026). Transforming Clinical Learning: Insights on Mobile Medical Imaging Applications Use Among Undergraduate Students. Saudi J. Med..

[75] Nassiri K., Akhloufi M.A. (2024). Recent Advances in Large Language Models for Healthcare. BioMedInformatics.

[76] Neha F., Bhati D., Shukla D.K., Amiruzzaman M. (2024). ChatGPT: Transforming Healthcare with AI. AI.

[77] Patel V.P., Jolkver E., Schwerk A. (2026). MedDiscover: A Domain-Specific Retrieval-Augmented Generation Framework for Evidence-Grounded Knowledge Extraction in Metabolomics. Comput. Struct. Biotechnol. J..

[78] Wang R., Wang J., Huang Y., Guo H., Pang J. (2026). A survey on medical multimodal retrieval-augmented generation (RAG). High-Confid. Comput..

[79] Wołk K. (2025). Evaluating Retrieval-Augmented Generation Variants for Clinical Decision Support: Hallucination Mitigation and Secure On-Premises Deployment. Electronics.

[80] Al-Anezi F.M. (2025). Challenges of Healthcare Systems in Saudi Arabia to Delivering Vision 2030: An Empirical Study From Healthcare Workers Perspectives. J. Healthc. Leadersh..

[81] Saudi Food and Drug Authority (2025). SFDA Leads Digital Transformation in Drug and Cosmetic Safety Regulation.

[82] Suleiman A.K., Ming L.C. (2025). Transforming healthcare: Saudi Arabia’s vision 2030 healthcare model. J. Pharm. Policy Pract..

[83] Aldosari H. (2026). Artificial Intelligence in Healthcare Administration and Clinical Informatics: A Critical Review and Governance Roadmap. Healthcare.

[84] Borat S., Chowdhury S. (2026). Artificial intelligence-driven clinical decision support systems for precision oncology: A comprehensive review. In Silico Res. Biomed..

[85] Elhaddad M., Hamam S. (2024). AI-Driven Clinical Decision Support Systems: An Ongoing Pursuit of Potential. Cureus.

[86] Fletcher E., Burns A., Wiering B., Lavu D., Shephard E., Hamilton W., Campbell J.L., Abel G. (2023). Workload and workflow implications associated with the use of electronic clinical decision support tools used by health professionals in general practice: A scoping review. BMC Prim. Care.

[87] Alshehri A.A., Abduljawad A.A. (2025). Impact of the Saudi Health Sector Transformation Program (SHSTP): A Mixed-Methods Evaluation of Patient-Centered Care and Digital Health Adoption. Healthcare.

