# Supplementary material for: Digital Health Revolution in Saudi Arabia: A Narrative Review of Healthcare Transformation
Source: Healthcare (Basel). 2026 Sep 2;14(17):2810. doi: 10.3390/healthcare14172810 (PMC13565072; doi:10.3390/healthcare14172810)
Supplement: Supplementary file 1 [file healthcare-14-02810-s001.zip › healthcare-4414767-supplementary.pdf]

Supplementary tables

Table S1. Characteristics of empirical studies included in the narrative review.

Citation	Country/ region	Healthcare setting	Technology/ domain	Study design	Sample/ data source	Comparator, if any	Outcomes assessed	Main reported findings	Key limitations	Saudi relevance
Alanazi & Alruthia (2026)	Saudi Arabia	500-bed tertiary referral center, Riyadh	CPOE and clinical documentation	Retrospective observational study	CPOE documentation records, 2015–2019; sample size not reported in manuscript	Documentation across years	Completeness of CPOE data; predictors of completeness	Completeness increased from 5.5% in 2015 to 26.0% in 2017 and 49.5% in 2019; 2019 was associated with higher completeness than 2015	Single-center study; documentation completeness is not a direct clinical outcome; residual confounding possible	Direct Saudi evidence on CPOE maturity, documentation quality, and pharmacist involvement

Alghamdi & Sayed (2026)	Saudi Arabia	Five public hospitals, Riyadh	EMR implementation	Concurrent mixed-methods study	61 hospital leaders involved in EMR implementation	Not applicable	Leadership and staff perceptions; implementation facilitators and barriers	Adequate infrastructure and positive staff perception were associated with implementation success; training, system issues, and time constraints were identified as barriers	Small sample; self-reported perceptions; leadership-focused sample; cross-sectional associations	Direct Saudi evidence on organizational readiness, leadership, and EMR implementation
Alanazi et al. (2024)	Saudi Arabia	Tertiary military hospital	Electronic prescribing	Retrospective pre–post study	29,554 admissions	Before versus after modifications to the electronic prescribing system	Inpatient prescribing-error rate	Overall prescribing errors decreased from 1.43% to 0.51% after system modifications	Non-randomized design; possible influence of concurrent changes; single hospital; department-level variation	Direct Saudi evidence on e-prescribing optimization and medication safety

El Mahalli et al. (2016)	Saudi Arabia	Three hospitals in the Eastern Province	Pharmacy-information systems and EHR integration	Cross-sectional system-performance assessment	Three hospitals; system-functionality assessment	Not applicable	Utilization and functionality of pharmacy information systems	Patient-administration functionality had highest use (56.8%); pharmacy-stock linkage had lowest use (9.1%); barcode-assisted medication administration and electronic adverse-event monitoring were not implemented	Older study; few hospitals; assessed system functions rather than patient outcomes; limited generalizability	Direct Saudi evidence on interoperability and pharmacy digital-functionality gaps
Al-Kahtani et al. (2022)	Saudi Arabia	Healthcare organizations assessed using HIMSS indicators	Digital-health transformation/ maturity	Cross-sectional analysis	Healthcare Information and Management Systems Society digital-health indicators; organizational sample size should be verified	Not applicable	Digital maturity, digital-health capability, and quality-related indicators	Described variation in digital-health transformation and implementation maturity across assessed domains	Cross-sectional design; maturity measures do not establish causal effects on patient outcomes	Nationally relevant evidence on Saudi digital-health maturity and readiness

Alajwari et al. (2022)	Saudi Arabia	Community/ citizen population during COVID-19	Telemedicine	Cross-sectional survey	Saudi citizens; sample size should be verified from original article	Not applicable	Knowledge and attitudes toward telemedicine	Reported public knowledge and attitudes toward telemedicine during the COVID-19 pandemic	Self-reported data; pandemic-specific context; not a clinical-effectivenes s study	Direct Saudi evidence on public acceptance and telemedicine readiness
Almalki et al. (2023)	Saudi Arabia	King Fahad Armed Forces Hospital	Automated medication-dispensing system	Quality-improvement/pr e–post project	Inpatient medication-dispensing process data; sample size should be verified	Before versus after automated -system implement ation	Medication turnaround time, drug consumption, returned medications	Reported an 83% reduction in medication turnaround time, 24% lower drug consumption, and 72% fewer returned medications	Single site; no concurrent control; operational outcomes may be influenced by workflow changes	Direct Saudi evidence on pharmacy automation and operational outcomes
Al Naam et al. (2022)	Saudi Arabia	Clinical laboratory	Total laboratory automation	Case study	Laboratory workforce and throughput data; sample size should be verified	Before versus after automatio n or comparati ve workflow assessmen t	Test throughput per worker; processing efficiency; workforce impact	Reported 1.4- to 3.7-fold increases in test throughput per worker and reduced manual processing burden	Single-site design; operational outcomes; limited direct patient-safety evidence	Direct Saudi evidence on laboratory automation and workforce efficiency

Alodhialah et al. (2024)	Saudi Arabia	Older-adult telehealth users	Telehealth adoption	Qualitative study	Saudi older adults; sample size should be verified	Not applicable	Barriers and facilitators to telehealth use	Identified technology access, digital literacy, support needs, and usability as important factors affecting telehealth adoption	Qualitative and context-specific; small sample likely; no comparative clinical outcomes	Direct Saudi evidence on telemedicine equity and older-adult digital inclusion
Alhur (2024)	Saudi Arabia	Healthcare/ EMR implementation context	EMR adoption	Descriptive or review-based report; verify article design	Not applicable	Not applicable	EMR adoption barriers and potential solutions	Identified training, user engagement, workflow, and technical support as important adoption considerations	Article design and empirical basis require verification; may not be eligible as an empirical study	Saudi contextual evidence on EMR adoption barriers
Alshamrani et al. (2025)	Saudi Arabia	Saudi tertiary-care centres	Automated dispensing cabinets	Observational study; exact design to verify	Sample size and number of centres should be verified	Not applicable	Adoption and effectiveness of ADCs; medication errors	Reported adoption and perceived effectiveness of ADCs in reducing medication errors	Design, sampling, and outcome definitions require verification; possible self-reporting bias	Direct Saudi evidence on pharmacy automation adoption

Alzahrani et al. (2024)	Saudi Arabia	Clinical pharmacy practice setting	Electronic prescribing	Observational study; exact design to verify	Sample size should be verified	Not applicable	Clinical-pharmacy practice and e-prescribing-related errors	Reported that approximately 8.5% of electronically prescribed medications required correction	Setting and denominator require verification; observational design; limited generalizability	Saudi evidence on new error types associated with electronic prescribing
Alanazi et al. (2022)	Saudi Arabia	Hospital medication-management setting	Automated drug-dispensing system	Observational or pre–post evaluation; exact design to verify	Sample size should be verified	Before versus after implementation, if applicable	Patient-safety outcomes related to automated dispensing	Reported that automated dispensing may improve medication-safety processes	Study design, sample, and reported effect estimates require verification	Direct Saudi evidence on automated dispensing and patient safety
Alshamrani (2025)	Saudi Arabia	Saudi healthcare organizations	EHR-enabled digital transformation	Operational/management study; exact design to verify	Sample size not stated in manuscript	Not applicable	Operational insights, interoperability, and EHR management implications	Discussed EHR-enabled digital transformation, interoperability, and management implications	Publication details and empirical data require verification	Saudi contextual evidence on EHR operations and interoperability

Atasoy, Greenwood & McCullough (2019)	United States/international evidence	Healthcare organizations	EHRs and digital patient care	Narrative/review article with empirical evidence synthesis	Literature reviewed; no single sample	Not applicable	Quality, utilization, and patient-safety effects of EHRs	Synthesized evidence on EHR effects, including clinical decision support and patient-safety mechanisms	Review design; heterogeneity across included studies; not Saudi-specific	Provides international contextual evidence only
Ayaad et al. (2019)	Country to verify	Healthcare institutions	EMRs	Comparative study	Sample size and setting should be verified	EMR-using hospitals versus paper-chart hospitals	Healthcare service quality, documentation, diagnostic efficiency, patient satisfaction	EMR-using hospitals reported higher service-quality measures than paper-based comparators	Country, sample, and causal design need verification; potential confounding	International/regional evidence requiring Saudi validation
Gatiti et al. (2021)	Developing-country settings; exact countries to verify	Hospitals	EHRs	Systematic review	Included studies; number should be verified	Not applicable	Hospital healthcare quality	Reported that EHRs can support communication, data access, documentation, and quality improvement	Review heterogeneity; variable EHR maturity and quality measures	International contextual evidence only

Upadhyay & Hu (2022)	Country/setting to verify	Healthcare clinicians	EHRs	Qualitative study	Clinician participants; sample size should be verified	Not applicable	Clinicians' experience of EHR effects on quality and safety	Identified real-time access, documentation, reminders, and workflow factors as mechanisms affecting quality and safety	Qualitative design; perceptions rather than measured outcomes; setting-specific	International contextual evidence only
Wani & Malhotra (2018)	United States	Healthcare organization s	Meaningful use of EHRs	Observational study	Study data and sample size should be verified	Organizati ons with different levels of meaningfu l EHR use	Patient outcomes and operational measures	Examined the association between meaningful EHR use and patient outcomes	Observation al design; potential confoundin g; health-system-specific setting	International evidence requiring Saudi validation
Uslu & Stausberg (2021)	International	Hospital care	EMRs	Literature review	Published hospital EMR studies	Not applicable	EMR value for hospital care	Synthesized evidence on data access, coordination, decision support, and hospital care outcomes	Review design; heterogeneo us intervention s and outcomes	International contextual evidence only
Porterfield , Engelbert & Coustasse (2014)	United States	Ambulatory care	Electronic prescribing	Narrative review	Published literature; no single sample	Not applicable	Prescribing efficiency and accuracy	Reported potential improvements in prescribing efficiency and accuracy	Older literature; narrative review; ambulatory-care focus	International contextual evidence only

Roumeliotis et al. (2019)	International	Hospital settings	Electronic prescribing strategies	Systematic review and meta-analysis	Included studies; total sample should be verified	Electronic prescribing versus usual or alternative prescribing processes	Medication errors, dosing errors, medication-related harm	Meta-analysis reported lower medication, dosing, and other medication-related errors with electronic prescribing strategies	Heterogeneity in interventions, settings, definitions, and study quality	Stronger international evidence, but transferability to Saudi settings requires evaluation
Camacho et al. (2024)	England	National Health Service	Digital transfer of prescription information	Modelling/economic and patient-safety analysis	National prescription data/modelled scenarios; exact data source to verify	Enabled versus non-enabled digital prescription transfer scenarios	Incorrect dosages and projected patient-safety benefits	Projected prevention of incorrect dosages and estimated 4–22 lives saved annually	Model-based projections rather than direct observed outcomes; country-specific infrastructure	International evidence, not directly transferable
Cassidy et al. (2023)	International	Community pharmacy	E-prescribing	Rapid scoping review	Included studies; number should be verified	Not applicable	Medication safety and prescribing errors	Most included studies reported lower prescription-error rates and improved medication safety	Scoping design; mixed study quality; community-pharmacy focus	International contextual evidence only

Shaikh et al. (2019)	International	Hospital settings	Electronic prescribing	Systematic review and narrative synthesis	Included studies; number should be verified	Not applicable	Healthcare professionals’ work practices	Identified workflow, communication, workload, and interaction effects, including potential reduced face-to-face interaction	Heterogeneous evidence; limited outcome standardization	International implementation evidence only
Rozenblum et al. (2020)	United States	Hospital/clinical medication-prescribing context	Machine-learning-enabled prescribing support	Clinical and cost evaluation	Sample size should be verified	Machine-learning system versus baseline prescribing processes	Prescribing-error identification, clinical relevance, costs	Reported identification and prevention of prescribing errors using a machine-learning system	Site-specific system; clinical integration and generalizability limitations	International evidence requiring local validation

Kammrath Betancor et al. (2025)	Germany	Ophthalmology practice and university hospital	Online appointment scheduling	Retrospective service evaluation	16,894 practice appointments and 81,173 hospital appointments over 20 months	Online versus non-online scheduling; effects of reminders and regular consultations	No-show rate, appointment utilization	Online scheduling was associated with lower no-show rates in the practice but higher rates in the hospital; reminders and regular consultations modified outcomes	Observational design; setting-specific; potential selection bias	International evidence; supports need for Saudi evaluation by setting
Zanaboni & Fagerlund (2020)	Norway	General-practice digital services	Online scheduling/e-consultation	Online survey	Patient respondents; sample size should be verified	Not applicable	Patient experience and use of digital services	Reported that many users viewed online scheduling as easier and more efficient than telephone scheduling	Online survey selection bias; self-reported outcomes; general-practice context	International evidence on user experience only

Shen, Yang & Sun (2019)	China	Shanghai public-health system	Online appointment diagnosis and hierarchical care	Case study/policy analysis	Health-system data; sample size should be verified	Before/after or contextual comparison not specified	Service allocation and public-health efficiency	Reported that online reservations may support referral of minor versus complex conditions across care levels	Case-study design; policy-specific setting; causal inference limited	International evidence requiring adaptation
Carini et al. (2021)	International	Patient-portal settings	Patient portals and appointment reminders	Systematic literature review	Included studies; number should be verified	Not applicable	Health outcomes, efficiency, and patient attitudes	Reported that integrated reminders can improve follow-up adherence and attendance	Heterogeneous interventions and outcomes; not Saudi-specific	International evidence on portal implementation
Ng'andu & Haabazoka (2024)	Zambia	Healthcare facilities	Digital health records and online appointments	Facility study; exact design to verify	Sample size should be verified	Digitalized versus non-digitalized processes, if applicable	Operational efficiency, patient flow, coordination	Reported improved patient flow and care coordination with digital records and appointment systems	Setting-specific; study design and data need verification	Evidence from a lower-/middle-income context; still requires Saudi validation

Dendere et al. (2019)	International	Inpatient healthcare settings	Patient portals and inpatient EMRs	Systematic review	Included studies; number should be verified	Not applicable	Patient engagement, documentation, quality, and safety	Reported that portals can facilitate engagement with inpatient EMRs and reduce communication/documentation gaps	Variable portal designs and study quality; mixed outcomes	International contextual evidence only
Huang, Yan & Yin (2021)	China	E-healthcare providers	Online–offline service integration	Quasi-natural experiment	Provider/service data; sample size should be verified	Before versus after integration or exposed versus comparison providers	Provider performance and operational outcomes	Evaluated effects of online–offline integration on provider outcomes	Country- and platform-specific context; study details require verification	International operational evidence only
Jones et al. (2024)	International	Radiation-oncology settings	AI and automation in treatment planning	Review or implementation-focused study; design to verify	Not applicable or included literature	Not applicable	Planning efficiency, precision, inter-observer variability	Reported potential improvements in planning efficiency and consistency	Evidence may be technology- and institution-specific; clinical outcome evidence limited	International evidence; local validation needed
Sharaf (2024)	Country/setting to verify	Hospital pharmacy	Prescription/pharmacy automation	Review article; not an empirical study	Not applicable	Not applicable	Medication safety and workflow efficiency	Discussed potential benefits of prescription automation	Should be excluded from empirical table; retain in reference list only	Not applicable

Meknassi Salime et al. (2025)	International	Hospital pharmacy	Pharmacy automation	Systematic review	Included studies; number should be verified	Not applicable	Drug tracking, workload, compatibility, training	Identified potential workflow and tracking benefits with compatibility and training challenges	Review design; heterogeneous technologies and outcomes	International contextual evidence only
Huang et al. (2024)	Taiwan	Healthcare administrative process	Robotic process automation for claims verification	Lean Six Sigma case study	Claims-verification process; sample size not reported in manuscript	Before versus after RPA implementation	Processing time and process-cycle efficiency	Saved 380 minutes of processing time; process-cycle efficiency improved from 69.07% to 95.54%	Single process and institution; administrative outcomes; no direct patient outcomes	International workflow evidence only
Chakhunashvili, Blommengren & Kullberg (2025)	Sweden	Karolinska University Hospital	Automated patient-reported experience measures	Implementation study	Patient-feedback process; sample size should be verified	Before versus after automation	Response volume and staff burden	Automated PREM process reportedly increased feedback responses tenfold	Single hospital; response quantity does not ensure representativeness or outcome improvement	International evidence on patient-feedback automation

Iftikhar et al. (2023)	Not applicable	Health-service context	ChatGPT-3 virtual assistant	Descriptive/pilot report; design to verify	Sample size not reported	Not applicable	Documentation, medication suggestions, communication	Reported potential utility of ChatGPT-3 as a virtual doctor	Likely non-validated; unclear methods; not suitable as strong effectiveness evidence	International/technical evidence only
Tu et al. (2023)	Taiwan	Critical-care units	Automated dispensing cabinets	Retrospective pre–post study	Medication-error reporting system; prescription and dispensing data; number of prescriptions/dispensations should be verified	Before versus after ADC adoption	Prescription, dispensing, administration errors and severity categories	Prescription errors fell from 3.03 to 1.75 per 100,000 prescriptions; dispensing errors fell from 3.87 to zero per 100,000 dispensations; administration errors fell from 0.046% to 0.026%	Incident-reporting data may underreport events; no concurrent control; single clinical context	International evidence relevant to Saudi critical-care pharmacy settings
Craswell et al. (2021)	Australia	New hospital	Distributed automated dispensing units	Qualitative study	Nursing and pharmacy staff; sample size should be verified	Not applicable	Workflow organization, staff experience, implementation barriers	Reported improved workflow organization and nurse satisfaction, alongside adaptation challenges	Qualitative, single-hospital study; no direct error-rate outcome	International implementation evidence only

Zheng et al. (2021)	International	Hospital settings	ADCs, BCMA, and closed-loop electronic medication management	Systematic review	Included studies; number should be verified	Not applicable	Work processes and medication safety	Identified possible safety and workflow improvements, but also usability problems, barcode issues, and override-related risks	Heterogeneous interventions and study designs; limited standardized outcomes	International evidence relevant to Saudi medication systems
Boyd & Chaffee (2019)	United States/international	Hospital pharmacy	Pharmacy automation and robotic systems	Critical review	Published literature	Not applicable	Safety, sustainability, and evaluation	Highlighted need for robust evaluation, financial sustainability, and operational planning	Review/opinion format; not an empirical study	Not applicable
Kaiser et al. (2023)	Germany	Medical rehabilitation	Health claims data for quality assurance	Study protocol combining prospective longitudinal and retrospective cohort methods	Claims data and planned cohorts; final sample not applicable in protocol	Not applicable	Mortality, readmissions, satisfaction, opioid prescribing, quality assurance	Proposed a cross-sectoral claims-data approach for quality monitoring	Protocol only; no completed results; should be separated from completed empirical studies	International methodological relevance only

Sharda et al. (2025)	International	General internal medicine	Machine learning for readmission prediction	Systematic review	Included studies; number should be verified	Not applicable	Hospital readmission prediction	Synthesized machine-learning models for predicting readmission	Review design; heterogeneity, risk of bias, and transportability concerns	International analytic evidence only
Erickson et al. (2020)	United States	Health-system policy context	Payment and delivery-system reform	Policy position paper	Not applicable	Not applicable	Healthcare delivery and payment reform	Discussed reform principles rather than a specific digital intervention effect	Not empirical; should be excluded from empirical table	Not applicable
Zouo & Olamijuwon (2024)	International	Healthcare finance context	Financial-data analytics	Narrative review	Not applicable	Not applicable	Efficiency and cost reduction	Discussed approaches to financial analytics in healthcare	Not empirical; exclude from empirical table	Not applicable
Dey et al. (2025)	United States	Healthcare billing/insurance	AI-based fraud detection	Review or technical study; design to verify	Dataset/sample size should be verified	Not applicable	Fraud detection and risk management	Reported potential for machine learning to identify anomalous claims	Study design, data source, and validation require verification	International technical evidence only
Nwosu (2024)	International	Healthcare operations	Business intelligence and data integration	Review/descriptive article	Not applicable	Not applicable	Operational costs and decision support	Discussed potential value of BI tools and data integration	Not empirical; exclude from empirical table	Not applicable

Ezeamii et al. (2024)	International	Telemedicine literature	Telemedicine	Narrative review	Not applicable	Not applicable	Access and patient outcomes	Reported potential for telemedicine to improve access and selected outcomes	Narrative review; heterogeneous evidence	Not empirical; exclude from empirical table
Palozzi, Schettini & Chirico (2020)	International	Rural healthcare	Telemedicine	Literature review	Included literature; number should be verified	Not applicable	Access to rural healthcare	Identified telemedicine as a possible means to improve rural access	Review design; variable contexts and interventions	International contextual evidence
Baughman et al. (2022)	United States	Large integrated health system	Primary-care telemedicine	Comparative observational study	Large system patient cohort; exact sample size should be verified	In-person versus telemedicine primary care	Quality-performance measures	Compared quality-performance measures across in-person and telemedicine care	Observational design; selection bias; integrated-US-system context	International comparative evidence; needs Saudi replication
Fieux et al. (2020)	France	ENT services during COVID-19	Telemedicine	Observational/service evaluation	ENT telemedicine consultations; sample size should be verified	Pandemic telemedicine versus usual practice, if applicable	Quality of care during COVID-19	Evaluated telemedicine contribution to ENT care continuity	Pandemic-specific; specialty-specific; short follow-up	International evidence requiring local context assessment

Society for Maternal-Fetal Medicine et al. (2023)	United States	Obstetric care	Telemedicine	Professional society statement	Not applicable	Not applicable	Quality and safety considerations	Provided practice considerations for telemedicine in obstetrics	Guidance document, not empirical evidence; exclude from empirical table	Not applicable
Khoong et al. (2022)	United States	Ambulatory telemedicine	Telemedicine expansion	Perspective/review or observational analysis; exact design to verify	Study sample should be verified	Not applicable	Patient-safety implications	Discussed patient-safety implications of rapid telemedicine expansion	Design and empirical basis require verification	International safety-context evidence
Neha, Bhati & Shukla (2025)	International	Healthcare AI/RAG literature	Retrieval-augmented generation	Systematic literature review	30 peer-reviewed studies	Not applicable	Diagnostic support, EHR summarization, medical question answering, RAG challenges	Identified early applications and limitations related to retrieval noise, domain shift, latency, and explainability	Many underlying studies were technical or pilot-based; limited real-world clinical validation	International emerging-AI evidence; Saudi implementation research is needed
Ibrahim et al. (2026)	International and evidence-synthesis based	Palliative-care/chronic-care context	Telemonitoring	Hybrid umbrella review of reviews and primary studies, reported using PRISMA 2020.	28 included studies; approximately 2,500 participants across primary empirical studies. Primary-study samples ranged from 40–450.	In some included primary studies. Comparators included usual in-person care, alternative telehealth, no intervention, or pre–post	Access, follow-up, and remote monitoring	Across 28 studies, nurse-led tele-palliative care was associated with better access to care, timely symptom assessment and monitoring, higher patient satisfaction, and some family-caregiver support. Evidence for reducing symptom severity and caregiver burden was mixed.	Considerable heterogeneity in interventions, populations, study designs, sample sizes, and outcome measures; variable methodological quality;	High practical relevance. It supports tele-palliative services led by nurses for patients who have difficulty attending specialist palliative-care clinics, including those in remote areas of Saudi

						assessment without controls.			substantial reliance on secondary evidence; many studies were from high-income countries.	Arabia.
Fahim et al. (2025)	Global healthcare context, including both high-resource and low-/middle-income settings; evidence from clinical, technological, and implementation literature.	Healthcare AI context	Artificial intelligence	Broad narrative/comprehensive literature review with a structured search of PubMed, Scopus, Web of Science, IEEE Xplore, and Google Scholar for English-language literature published 2015–2025.	Not reported	Not applicable	AI applications in diagnosis, treatment, and safety	AI has potential to strengthen diagnostics, treatment planning, patient monitoring, drug discovery, predictive analytics, telemedicine, and healthcare efficiency. The review emphasizes AI's potential to address access, workforce, cost, and quality challenges.	Narrative-review design means its conclusions depend on the breadth and quality of cited literature rather than pooled effect estimates. It does not provide a single standardized comparative evaluation or a fixed included-study sample.	High strategic relevance. The paper aligns with Saudi healthcare digital-transformation priorities
Mariano et al. (2025)	Saudi Arabia	Healthcare AI context	Artificial intelligence	Descriptive correlational cross-sectional study.	349 participants.	Not applicable	AI in healthcare	The study provides Saudi baseline evidence on AI knowledge, attitudes, and practices among nurses, nursing faculty, and students. It identifies the need to strengthen AI	Cross-sectional design prevents causal inference; online convenience sampling may introduce	Direct relevance. This is the most locally applicable study for identifying nursing AI-readiness gaps in Saudi Arabia.

								education, practical competence, and organizational readiness across nursing education and practice.	selection bias; self-reported responses may not reflect actual AI competence or use.	
Nassiri & Akhloufi (2024)	Healthcare and biomedical AI literature;	Healthcare data/AI context	AI and NLP	Narrative/scoping-style review of recent LLM advances in healthcare.	Not applicable	No formal comparator.	AI analysis of structured and unstructured data	LLMs may assist with clinical documentation, medical education, medical question answering, information retrieval, and decision-support tasks. The review also stresses that healthcare deployment must be evidence-grounded and carefully supervised.	Review-level evidence rather than a clinical effectiveness trial; rapid model changes can make findings quickly outdated. Core risks include hallucinations, inaccurate or non-transparent output, limited clinical reasoning, bias, privacy concerns, and insufficient real-world validation.	High but conditional relevance. LLMs could support Arabic/English documentation, patient education, clinical knowledge retrieval, and healthcare training in Saudi Arabia.

Omer et al. (2026)	Saudi Arabia	Healthcare AI context	AI/LLMs	Descriptive cross-sectional design.	490 participants.	Observational group comparisons. Students with versus without installed imaging apps were compared	AI analysis of clinical data	Students generally regarded AI apps as useful supplements—not universal replacements—for logbooks and pocketbooks.	Descriptive cross-sectional design cannot establish that apps caused better skills; convenience sampling and self-reporting increase selection and response bias; practical competence was perceived rather than objectively tested; participating students were disproportionately from private institutions; app content and quality were not independently standardized.	Direct relevance. It provides Saudi evidence to support incorporating carefully selected AI mobile imaging apps into radiology curricula and clinical training.

Patel et al. (2026)	Computational/benchmarked setting	Healthcare AI context	RAG	Reproducible computational RAG-development and benchmark evaluation study.	Gold set: 10 papers and 30 expert-curated QA pairs; Silver set: 100 papers and approximately 600 synthetic QA pairs—300 for Ada and 300 for MedCPT. Gold cross-system analyses used 20 questions with outputs available for all systems.	Yes. Compared MedCPT embeddings + GPT-4o versus Ada embeddings + GPT-4o, and versus a GPT-4o non-RAG baseline.	RAG architecture and applications	Retrieval augmentation improved evidence grounding compared with GPT-4o without retrieval. On the synthetic silver benchmark, the domain-specific MedCPT retriever showed higher answer correctness than Ada embeddings, while faithfulness, context recall, context precision, and relevance were broadly comparable. The study provides open code, document lists, and evaluation materials for reproducibility.	The Gold set was small (10 papers; 30 expert-curated QA pairs, with a 20-question subset for cross-system comparison). The Silver dataset used machine-generated QA pairs and is not a substitute for expert-validated clinical correctness.	Moderate-to-high research relevance. Saudi universities, laboratories, and health systems could adapt the framework for Arabic/English evidence retrieval in areas such as diabetes, obesity, inherited metabolic disorders, genomics, or local clinical guidelines.
Wang et al. (2026)	Medical multimodal-RAG research literature, covering applications that combine text with medical images and potentially other clinical modalities.	EHR context	RAG for EHR summarization	Survey/review article.	Not applicable	No formal experimental comparison by the review itself.	Retrieval of clinical history and summary generation	The survey maps the medical multimodal-RAG landscape, including methods that combine text with images and other medical data. It identifies potential applications in radiology, medical visual question answering, clinical information retrieval, and evidence-grounded multimodal decision support.	As a survey, it does not establish the clinical effectiveness of one system. The field has heterogeneous tasks, datasets, metrics, and implementations.	High future relevance. Multimodal RAG could be particularly useful in Saudi radiology, pathology, and clinical education because it can link imaging with reports, guidelines, and evidence.

Neha et al. (2024)	ChatGPT: Transforming Healthcare with AI	Broad healthcare settings: patient education and engagement, symptom assessment, triage, clinical decision support, diagnostics, specialist care, medical education, research, clinical documentation, administration, and emergency-response contexts.	ChatGPT and related generative-AI/large-language-model technologies in healthcare. The paper addresses natural-language processing, clinical decision support, health communication, medical-information retrieval, documentation, diagnostic support, and specialty applications.	Broad systematic/narrative literature review. It synthesizes prior evaluations and comparative studies of ChatGPT across organ systems, clinical specialties, education, administration, and patient-facing uses. It is not a primary clinical trial or a single empirical study.	Secondary published literature. There is no single patient sample, clinical dataset, or pooled participant number for the review itself. The review includes diverse prior studies using medical questions, clinical reports, examination questions, patient-information prompts, expert assessments, imaging reports, and specialty-specific datasets.	No single comparator for the review. Included studies variously compared ChatGPT versions (e.g., GPT-3.5, GPT-4, GPT-4o), other AI tools/search engines, clinical guidelines, and/or healthcare professionals.	Accuracy, completeness, reliability, readability, safety, guideline concordance, diagnostic performance, educational usefulness, empathy, patient-information quality, clinical decision-support value, response consistency, and ethical/implementation risks.	ChatGPT shows potential to improve patient engagement, provide accessible health information, assist history-taking and symptom assessment, support clinical documentation, enhance education, and contribute to decision support. Its performance can be strong for selected factual or educational tasks—for example, GPT-4 achieved 100% accuracy in one heart-failure question evaluation and 90% on a dermatology examination assessment.	The review aggregates highly heterogeneous studies, ChatGPT versions, prompts, methods, outcomes, clinical topics, and comparators, so a single effect estimate is not possible. ChatGPT can generate inaccurate, incomplete, outdated, biased, or non-verifiable information; it may hallucinate, lack context-sensitive clinical judgment.	High strategic relevance, but indirect evidence. The study supports potential Saudi applications in Arabic/English patient education, appointment and triage support, clinical documentation, medical and nursing education, literature summarization, and administrative workflow improvement.

Table S2. Characteristics and strength of evidence on digital transformation interventions and outcomes.

Intervention	Main outcome(s)	Typical evidence in this review	Interpretation to use
EHRs/EMRs and CPOE	Documentation quality, workflow, medication safety	Observational studies, retrospective studies, mixed-methods evaluations, systematic reviews	Associated with improved documentation, coordination, or selected safety outcomes.
E-prescribing	Prescribing and medication errors	Systematic reviews/meta-analyses, retrospective pre–post studies	Evidence suggests that e-prescribing can reduce prescribing errors; effects depend on system design and implementation."
Automated dispensing cabinets/BCMA	Medication-dispensing and administration errors	Before-and-after studies, quality-improvement projects, systematic reviews	"Associated with lower reported error rates in some settings.
Telemedicine	Access, satisfaction, continuity, service use	Observational studies, surveys, qualitative studies, systematic reviews	May improve access and patient-reported satisfaction; clinical and safety outcomes vary by condition and care model.
Online appointment systems	Access, no-shows, waiting time	Retrospective service evaluations and observational studies	May improve scheduling efficiency; effects on no-show rates may differ by setting.
Clinical/administrative automation	Turnaround time, workload, process efficiency	Case studies, pilot projects, pre–post studies	Shows potential to improve selected workflow measures; evidence on patient outcomes is limited.
Claims/financial analytics	Processing efficiency, claim denials, operational measures	Case studies, secondary reports, descriptive evidence	May support operational efficiency; robust evidence on patient safety and ROI remains limited.
Economic outcomes/ROI	Costs, savings, return on investment	Secondary reports, modelling studies, case studies, local business evaluations	Reported savings and ROI should be interpreted cautiously because methods, cost components, and settings vary.
